# Long-Lived Triplets from Singlet Fission in Pentacene-Decorated Helical Supramolecular Polymers

**DOI:** 10.1021/jacs.4c09844

**Published:** 2024-10-08

**Authors:** Giulia Lavarda, Ashish Sharma, Marko Beslać, Stef A. H. Jansen, Stefan C. J. Meskers, Richard H. Friend, Akshay Rao, E. W. Meijer

**Affiliations:** † Institute for Complex Molecular Systems and Laboratory of Macromolecular and Organic Chemistry, Eindhoven University of Technology, 5600 MB Eindhoven, The Netherlands; ‡ Department of Physics, Cavendish Laboratory, University of Cambridge, Cambridge CB30HE, United Kingdom

## Abstract

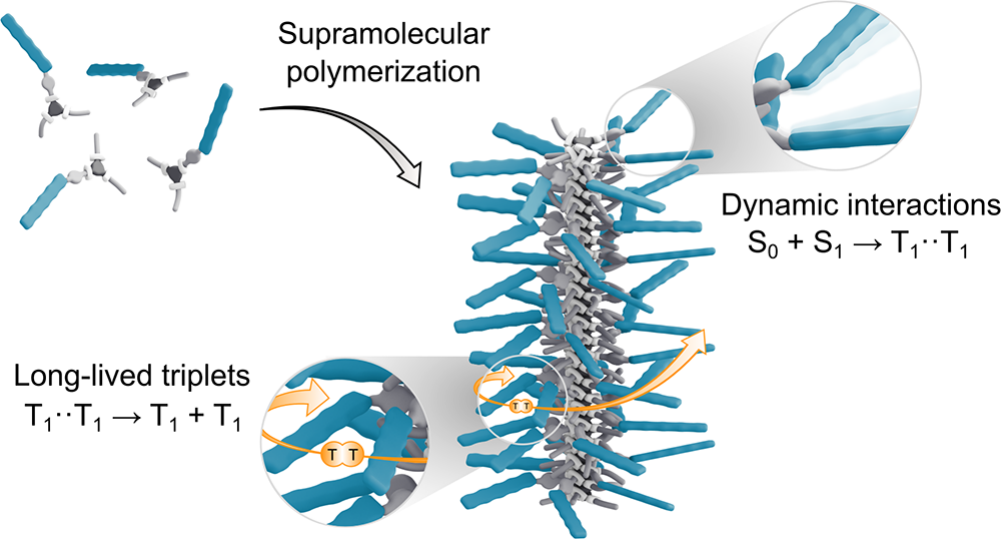

Singlet fission (SF), which involves the conversion of a singlet excited state into two triplet excitons, holds great potential to boost the efficiency of photovoltaics. However, losses due to triplet–triplet annihilation hamper the efficient harvesting of SF-generated triplet excitons, which limits an effective implementation in solar energy conversion schemes. A fundamental understanding of the underlying structure–property relationships is thus crucial to define design principles for cutting-edge SF materials, yet it remains elusive. Herein, we harness helical supramolecular polymers decorated with pentacene side groups to elucidate intermolecular SF dynamics in solution and promote the formation of long-lived mobile triplets. By leveraging the hydrogen bonding-driven assembly of benzene-1,3,5-tricarboxamide (BTA) cores into one-dimensional scaffolds, we direct the organization of appended pentacene motifs into long-range ordered helical frameworks. Dynamic interactions between weakly coupled SF pendants mediate singlet conversion within hundreds of picoseconds, affording triplet quantum yields well above 100%. Moreover, analysis of triplet dynamics with a Monte Carlo simulation model reveals that triplet diffusion along the supramolecular fibers is favored over annihilation, resulting in independent triplets exhibiting considerably slow decay on the time scale of tens of microseconds. The molecular packing within the assembly is tuned by subtle changes in monomer design to increase the rate and efficiency of SF while ensuring exceptionally long-lived mobile triplets, allowing to maintain triplet quantum yields exceeding 100% for at least 100 ns. This work opens new opportunities to exploit self-assembled supramolecular polymers as functional templates to achieve long-lived SF-generated triplets.

## Introduction

Singlet fission (SF)^1,2^ holds great potential as a down-conversion strategy to reduce energy losses and boost the efficiency of solar cells beyond the 33% thermodynamic limit.^3−6^ In its simplest description, this process involves the interaction of a chromophore in the singlet excited state ^1^(S_1_) with a neighboring ground-state chromophore ^1^(S_0_) to afford two independent triplet excitons ^3^(T_1_) via a correlated triplet pair ^1^(T_1_T_1_).^1,7−10^ To effectively implement SF in photovoltaics, several challenges beyond fast singlet conversion must be addressed.^5,6,11^ Ultrafast geminate and nongeminate annihilation of triplet excitons back to the ground state stands out as a critical bottleneck, preventing the formation of long-lived triplets and thus hindering the efficient harvesting of SF-generated triplet excitons.^12,13^ Therefore, a fundamental understanding of the factors that balance the rates and efficiencies of SF and backward triplet–triplet annihilation is crucial for the development of design principles for cutting-edge SF materials.

In this regard, several studies have been conducted to explore the influence of chromophore arrangement in films,^14^ nanoparticles,^15^ covalently linked dimers^16−18^ and macromolecules,^19^ among others, with pentacene (Pnc) being employed as prototypical SF system. Although the underlying principles remain elusive, the strength of interchromophoric interactions and long-range order appear to be instrumental in regulating triplet dynamics.^20−22^ Specifically, the electronic coupling between individual SF chromophores must be strong enough for singlet conversion to be fast and efficient, yet weak enough to ensure the independent behavior of the resulting triplets.^20,21,23^ Solid-state systems further favor the efficient formation of free triplets by providing pathways for triplet diffusion, which promotes spatial separation of triplet excitons.^14,20^ However, their inherent complexity hampers the elucidation of structure–property relationships governing intermolecular SF. On the other hand, spatial confinement and strong interchromophoric interactions in intramolecular SF systems hinder the formation of independent triplet excitons, favoring triplet pair annihilation to the ground state.^16,24,25^ Persistent triplets in covalent SF architectures have been achieved in isolated cases by reducing electronic coupling with alkyl spacers,^26^ or promoting exciton migration through energy gradients^27^ or nonconjugated oligomeric^28^ and macromolecular frameworks.^29^

Considering the aforementioned, we envisioned that Pnc-decorated supramolecular polymers with ordered helical chirality could serve as model systems to gain insight into intermolecular SF in solution and promote the formation of long-lived mobile triplets. Leveraging directional secondary interactions between monomeric units equipped with SF functionalities provides a strategy to mitigate the strength of interchromophoric interactions while imparting supramolecular order reminiscent of crystalline materials.^30,31^ In addition, the dynamic and reversible nature of supramolecular polymers offers the intriguing possibility to trigger the assembly and activate SF under dilute conditions by tailoring external stimuli such as temperature and solvent polarity. Remarkably, SF within one-dimensional aggregates in solution has rarely been addressed in the literature, with studies limited to stacked polyene systems.^32,33^

Herein, we harness the cooperative assembly of benzene-1,3,5-tricarboxamide (BTA) cores into chiral stacked supramolecular scaffolds to guide the organization of appended Pnc chromophores into long-range ordered helical frameworks. Dynamic interactions between weakly coupled photoactive pendants enable SF within hundreds of picoseconds. Moreover, favored triplet diffusion along the supramolecular fibers results in independent triplets that decay on a remarkably long time scale of tens of microseconds. Subtle modifications in the monomeric structure are exploited to tune the molecular packing within the assembly and the resulting excited state kinetics, affording triplet yields exceeding 100% for at least 100 ns.

## Results and Discussion

### Monomer Design and Synthesis

**Figure 1:**
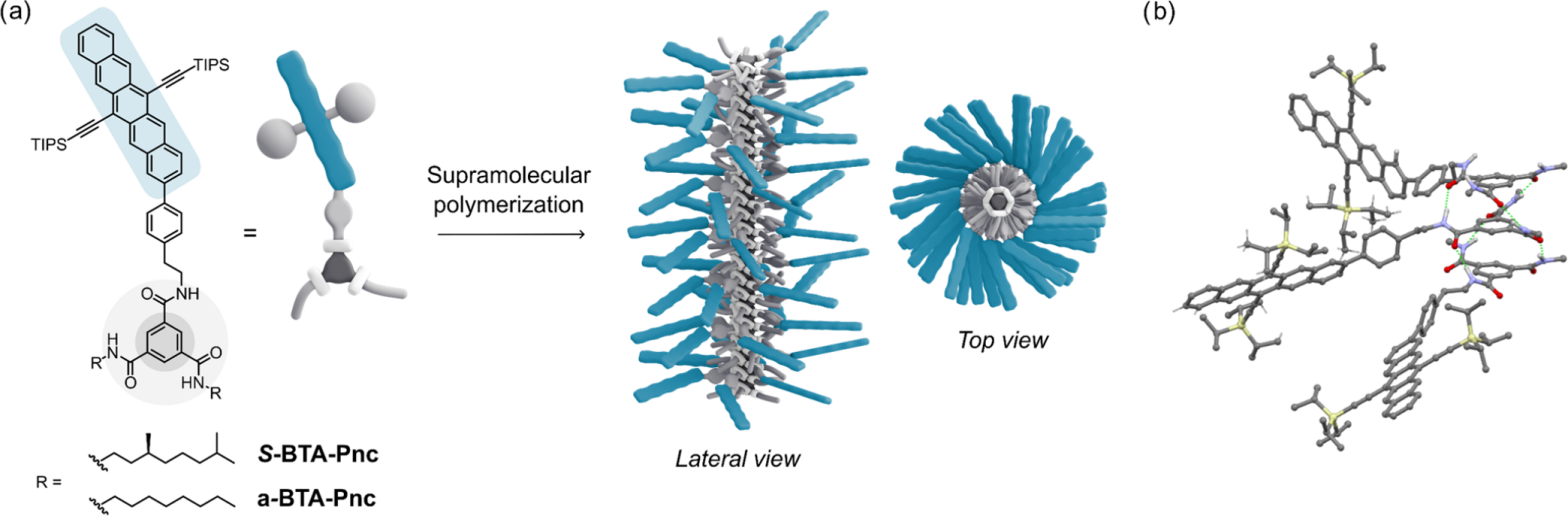
(a) Chemical structure of ***S*****-BTA-Pnc** and **a-BTA-Pnc** monomers and cartoon depicting self-assembly into helical supramolecular polymers. (b) Molecular modeling of BTA-Pnc supramolecular oligomers showing 3-fold hydrogen-bonding interactions between monomers.

To promote assembly into one-dimensional supramolecular polymers and enable SF, we designed a building block (***S*****-BTA-Pnc**, Figure 1a) comprising a BTA core functionalized at one of the amide groups with a Pnc moiety. An ethyl linker provides conformational flexibility to the Pnc pendant. The ability of BTA to form helical stacks via 3-fold hydrogen bonding is well-documented.^34^ On the other hand, triisopropylsilyl (TIPS)-Pnc represents a prototypical chromophore for fundamental investigation on SF due to its favorable excited-state energetics.^16,35,36^ A monofunctionalization pattern of the BTA core was chosen to prevent intramolecular SF, and the remaining amides were equipped with solubilizing (*S*)-3,7-dimethyloctyl substituents. To gain insight into the impact of monomer design on SF dynamics, we devised an analogous derivative featuring linear *n*-octyl side chains (**a-BTA-Pnc**, Figure 1a). Both target monomers were synthesized by COMU-mediated amide coupling between the amine-functionalized Pnc precursor (**Pnc-NH**_**2**_) and the corresponding **a-BTA-COOH** or ***S*****-BTA-COOH** precursor.^37,38^ The final compounds were purified by common laboratory techniques and fully characterized by ^1^H NMR, ^13^C NMR, and mass spectrometry (further details in SI).

### Self-Assembly Studies

**Figure 2:**
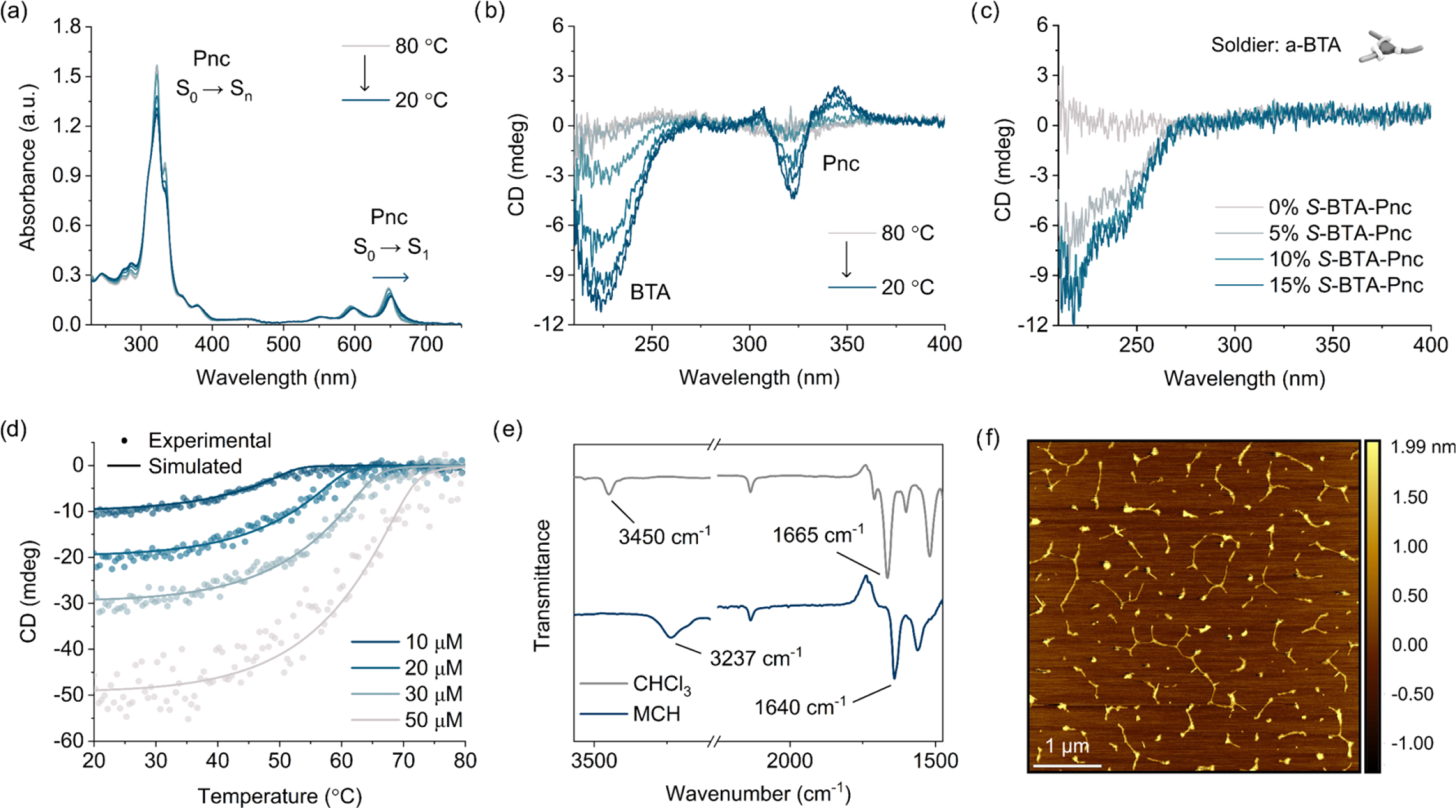
Supramolecular polymerization of ***S*****-BTA-Pnc** in MCH. (a) VT-Absorption and (b) VT-CD spectra of 10 μM ***S*****-BTA-Pnc** in MCH measured upon controlled cooling from 80 to 20 °C at a rate of 1 °C min^–1^ (optical path length: 10 mm). The arrow in (a) indicates the bathochromic shift of the high-wavelength Pnc absorption. (c) CD spectra of 10 μM ***S*****-BTA-Pnc**/**a-BTA** copolymers in MCH at 20 °C in the presence of different molar ratios of ***S*****-BTA-Pnc** sergeant (optical path length: 10 mm). (d) Experimental CD cooling curves (circles) of 10, 20, 30, and 50 μM ***S*****-BTA-Pnc** in MCH monitored at 224 nm obtained by controlled cooling from 80 to 20 °C at a rate of 1 °C min^–1^ (optical path length: 10 mm) and optimized fit (lines) of the mass-balance model for cooperative supramolecular polymerization to the experimental data. (e) FT-IR spectra of 0.5 mM ***S*****-BTA-Pnc** in chloroform and MCH. (f) AFM image of a spin-coated sample prepared by deposition of 10 μM ***S*****-BTA-Pnc** in MCH on freshly cleaved mica.

The self-assembly behavior of ***S*****-BTA-Pnc** in solution was investigated by UV–vis absorption and circular dichroism (CD) spectroscopy. UV–vis spectra are dominated by the characteristic absorption pattern of the Pnc chromophore, including a complex feature in the 300–400 nm region along with a less intense band spanning 500–700 nm and exhibiting vibrational fine structure (Figures 2a and S9a).^36^ At room temperature, the compound is molecularly dissolved in chloroform in the range of 10 to 200 μM, as indicated by the linear concentration dependence of absorbance and the lack of CD response (Figure S9).

On the other hand, distinct changes in the variable temperature (VT) spectra of 10 μM ***S*****-BTA-Pnc** solutions in methylcyclohexane (MCH) provide clear evidence for the formation of supramolecular polymers with a preferred helicity (Figure 2a,b). Whereas the absence of any CD signal at 80 °C indicates that the compound is present in solution as a monomer, controlled cooling to 20 °C leads to the appearance and rapid increase of a negative Cotton effect in the BTA absorption region (*i.e*. < 270 nm). This signal strongly resembles the characteristic CD band of *N*,*N′*,*N*″-tris((*S*)-3,7-dimethyloctyl)benzene-1,3,5-tricarboxamide (***S*****-BTA**) assemblies and is attributed to a helical columnar arrangement of the BTA cores in ***S*****-BTA-Pnc**.^34,39^

Interestingly, an additional bisignate Cotton effect is observed in the wavelength range of the high-energy Pnc absorption, evidencing a chiral organization of the pendant motifs (Figure 2b).^40^ The lower (about 15-fold) molar ellipticity compared to that reported for stacked Pnc aggregates suggests a significantly weaker coupling between chromophores.^41^ On the other hand, the low-energy Pnc absorption remains optically inactive, but exhibits a bathochromic shift (namely 5 nm) upon cooling (Figure 2a). The latter is generally diagnostic for J-type exciton coupling in staggered architectures, with the modest magnitude of the shift providing further evidence for weak interchromophoric interactions.^42^ Overall, these results can be rationalized on the basis of a spiral staircase arrangement of Pnc pendants in the form of a one-handed exohelix around the stacked BTA backbone (Figures 1 and S30).

To validate the cooperativity of the assembly process, we investigated whether small amounts (5 to 15% mol/mol) of ***S*****-BTA-Pnc** could bias the helicity of *N*,*N′*,*N″*-tris(*n*-octyl)benzene-1,3,5-tricarboxamide (**a-BTA**) stacks. This experiment is based on the “sergeant-and-soldiers” principle observed for cooperative assemblies of *C*_3_-symmetric discotic molecules, where chiral sergeants dictate the copolymer handedness when assembled with achiral soldiers.^39,43,44^ The nonlinear dependence of the CD intensity at 224 nm of 10 μM ***S*****-BTA-Pnc/a-BTA** mixtures on the molar ratio of chiral sergeant confirms the capability of ***S*****-BTA-Pnc** to dictate the helical sense of BTA stacks (Figure 2c). Notably, no Cotton effect is observed in the Pnc absorption region for the explored range of ***S*****-BTA-Pnc** ratios. This further confirms that ordered domains of close packing of the Pnc pendants are required for the emergence of a distinct Pnc CD signal as that observed for solutions of ***S*****-BTA-Pnc** homopolymers. The nonsigmoidal shape of the CD cooling curves recorded at 224 nm for ***S*****-BTA-Pnc** in MCH is illustrative of a nucleation-elongation mechanism governing the assembly (Figure 2d). Fitting a cooperative mass-balance model to the experimental data yields the thermodynamic parameters shown in Table S1 (further details in SI, section 3.2).^45^ A lower nucleation penalty (NP = 16.85 kJ mol^–1^) is observed for ***S*****-BTA-Pnc** monomers compared to ***S*****-BTA**, which can be explained by the steric influence of the bulky TIPS-acene substituent.^34^

The addition of 0.5% (*v*/*v*) MeOH as a scavenger to 10 μM ***S*****-BTA-Pnc** in MCH at the end of the cooling ramp results in the complete loss of chiroptical response, highlighting the key role of hydrogen-bonding interactions between amides in driving the formation of the long-range ordered helical aggregates (Figure S10). The formation of hydrogen-bonded assemblies of ***S*****-BTA-Pnc** in MCH was confirmed by Fourier transform infrared (FT-IR) spectroscopy (Figure 2e).^46^ Whereas in chloroform, where the compound is molecularly dissolved, the N–H and carbonyl stretching vibrations are found at 3450 and 1665 cm^–1^, respectively, MCH solutions exhibit the typical shift at lower wavenumbers (namely, 3237 and 1640 cm^–1^) of hydrogen-bonded amides. On the other hand, atomic force microscopy (AFM) of spin-coated samples prepared by deposition of 10 μM solutions of ***S*****-BTA-Pnc** in MCH on freshly cleaved mica revealed the formation of sub-μm long one-dimensional structures with height profile (∼2 nm) compatible with that expected for single fibers (Figures 2f, S15, and S16).

The observed assembly behavior of ***S*****-BTA-Pnc**, guided by the formation of a stacked BTA scaffold, is intriguing and not trivially predictable. Not surprisingly, the supramolecular polymerization of BTA monomers asymmetrically functionalized with π-extended moieties has received little attention in the literature. Indeed, π–π stacking between conjugated substituents may outcompete interactions between BTA cores. Here, apparently, hydrogen bonds between amides are not disturbed due to the steric hindrance of the bulky TIPS groups ensuring weak Pnc-Pnc interactions, and together they play a crucial role in dictating the molecular arrangement within the assembly.

The self-assembly study of **a-BTA-Pnc** in MCH revealed a similar behavior as the branched analog (Figures S11–13 and S17). As expected, no Cotton effect is detected for 10 μM solutions of **a-BTA-Pnc** due to the formation of equal amounts of *M* and *P* helices (Figure S11b). The amplification of asymmetry observed in the presence of low ratios of ***S*****-BTA** as chiral sergeant confirms the ability of **a-BTA-Pnc** to (co-)assemble into helical stacks (Figure S12a). In particular, a 10% mole fraction of ***S*****-BTA** is sufficient to fully bias the helicity of the copolymers. Similar results are obtained using ***S*****-BTA-Pnc** as a chiral dopant (Figure S12b). Of note is the different BTA CD profile of ***S*****-BTA-Pnc** homopolymers (single Cotton effect minimizing at 224 nm, Figure 2b) and **a-BTA-Pnc/S-BTA** or **a-BTA-Pnc/S-BTA-Pnc** copolymers (double Cotton effect with a minimum at 213 nm and a shoulder around 250 nm, Figure S12), being diagnostic of different molecular packing within the stacks. These CD features resemble those of ***S*****-BTA** homopolymers and **a-BTA** copolymers, respectively (Figures S12a and 2c). It has been proposed that the dihedral angle (θ) between the C=O and central benzene planes is different for a stack of primarily chiral BTA monomers (θ = 45°) versus a stack of primarily achiral BTA monomers (θ = 35°).^47^ Hence, the characteristic CD profile of the latter indicates a smaller inter-ring distance compared to that found in ***S*****-BTA** stacks, suggesting a tighter packing of BTA cores within the supramolecular backbone of **a-BTA-Pnc** than in ***S*****-BTA-Pnc**.^48^

### Photophysics

After elucidating the self-assembly behavior of ***S*****-BTA-Pnc** and **a-BTA-Pnc**, we investigated the excited-state features of their supramolecular polymers in solution to explore whether Pnc pendants are capable of undergoing SF. To establish a baseline for comparison, we also examined equimolar monomer solutions, in which SF is not expected to occur.

**Figure 3:**
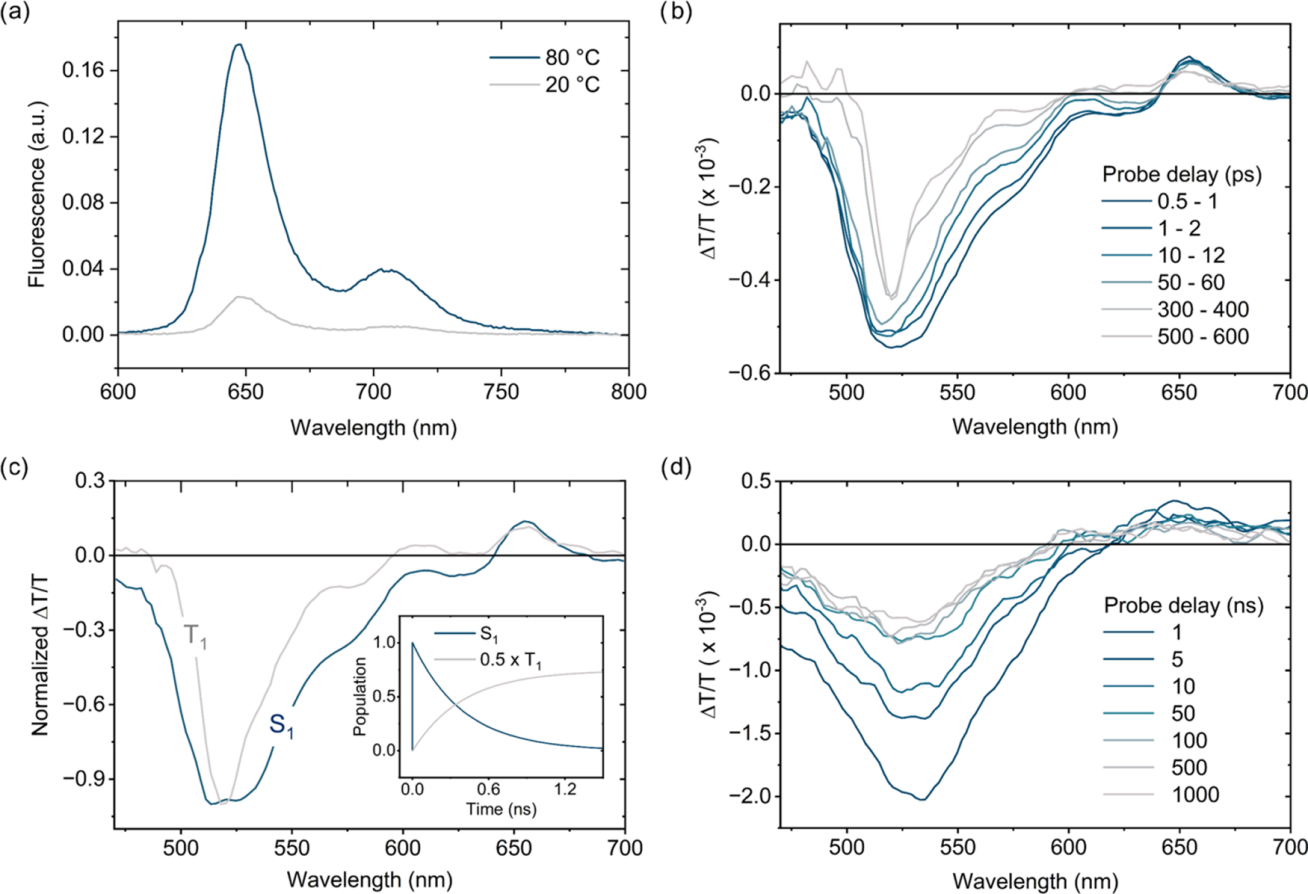
Photophysical characterization of ***S*****-BTA-Pnc** solutions. (a) Steady-state emission spectra (λ_ex_ = 321 nm) of 10 μM ***S*****-BTA-Pnc** in MCH at 80 °C (monomerically dissolved state) and 20 °C (assembled state). (b) TA spectra in the ps time scale of 10 μM ***S*****-BTA-Pnc** in MCH at room temperature (assembled state, λ_ex_ = 660 nm). (c) Evolution associated species spectra extracted by global fitting of experimental data in (b). Inset in figure (c) shows the time evolution of S_1_ and T_1_ species obtained through global fitting. (d) TA spectra in the ns time scale of 10 μM ***S*****-BTA-Pnc** in MCH at room temperature (assembled state, λ_ex_ = 355 nm).

Steady-state and time-resolved fluorescence measurements were thus performed on 10 μM ***S*****-BTA-Pnc** in MCH at 80 °C (monomerically dissolved state) and 20 °C (assembled state). Photoexcitation of ***S*****-BTA-Pnc** monomers at 321 nm yields the characteristic vibrationally structured emission previously reported for dilute solutions of TIPS-Pnc, with a fluorescence lifetime of 11.7 ns (Figure 3a).^36^ When solutions of ***S*****-BTA-Pnc** supramolecular polymers are examined, a dramatic quenching (nearly 90%) of the Pnc emission is observed. The absence of significant changes in the emission profile, which retains the fine structure, rules out contributions from excimer species.^49^ Time-correlated single photon counting (TCSPC) measurements on solutions of assembled ***S*****-BTA-Pnc** revealed a biexponential decay, with a short-lived component of 0.7 ns and a long-lived component of 13.6 ns. The latter resembles the monoexponential time constant determined for the monomerically dissolved species. Overall, these results indicate the activation of a fast, nonradiative channel for singlet decay in the assembly, for which SF may provide a rationale. We speculate that the ns-lived component at 20 °C, as well as the residual steady-state emission, may be partially attributed to the radiative decay of monomeric species still present in solution. To support this hypothesis, we estimated the amount of free monomer in 10 μM ***S*****-BTA-Pnc** in MCH by fitting the cooperative mass-balance model to the CD cooling curves, which yielded a molar ratio equal to 4% of the nominal concentration (Figure S14).^45^

To gain deeper insight into the excited state dynamics of monomerically dissolved and assembled species, picosecond (ps) and nanosecond (ns) transient absorption (TA) spectroscopy measurements were performed with 10 μM solutions of ***S*****-BTA-Pnc** at room temperature in either chloroform or MCH, respectively. The transient signatures of Pnc singlet and triplet excited states have been extensively investigated in the literature, allowing a straightforward assignment of photoinduced absorptions (PIAs) in the recorded TA spectra.^17,18,36^

In both solvents, photoexcitation at 660 nm leads to the immediate population of the Pnc singlet excited state (S_1_), as evidenced by the prompt appearance of fingerprint features in ps TA experiments–namely, a transient ground-state bleaching (GSB) at 650 nm and a broad PIA with a minimum around 520–540 nm (Figures 3b and S19a,b). However, substantial differences in the spectral evolution of the Pnc singlet PIA are observed when comparing MCH and chloroform solutions. For the latter (monomerically dissolved state), no significant spectral changes are detected in the ps regime, whereas a predominantly monoexponential decay (τ = 10.5 ± 0.5 ns) to the ground state is observed at longer probe delays (Figures S19 and S20). These findings are consistent with those from steady-state and time-resolved fluorescence assays on MCH solutions at 80 °C, pointing to an emissive decay of Pnc S_1_ for monomeric ***S*****-BTA-Pnc**. For ***S*****-BTA-Pnc** supramolecular polymer solutions in MCH, a different deactivation scenario is operative. In this case, the Pnc S_1_ PIA undergoes a slight blue-shift within hundreds of picoseconds, and a recovery of GSB in the 600–620 nm region is observed (Figure 3b). A remarkably slow decay of the shifted feature, with no change in spectral shape, is then observed on the time scale of tens of microseconds (Figure 3d).

Quantitative analysis of the spectral changes observed in the ps time scale was performed by global fitting of the TA data set (more details in SI, section 4.4). The global fit reveals that only two species are sufficient to explain the ps-TA data, *i.e*., photoexcited singlets which convert into a species reminiscent of Pnc triplets (T_1_) (Figure 3c).^17,18,36,40^ Thus, the observed spectral changes in the ps time scale are attributed to SF. The rate of SF obtained from the global fit (1.88 ns^–1^) is similar to the fast decay (1.42 ns^–1^) observed in TCPSC measurements of ***S*****-BTA-Pnc** solutions in MCH at 20 °C. The rapid decay of the Pnc S_1_ features and the simultaneous increase of the triplets confirm that SF is the *modus operandi* of triplet exciton generation within the fibers. Triplet quantum yield (TQY) quantifications further support that SF is the dominant decay mechanism of Pnc singlet excitons in ***S*****-BTA-Pnc** supramolecular polymers, with values exceeding 100% (Table S2, see section 4.3 in SI for further details). To the best of our knowledge, the exploitation of noncovalent polymeric templates to enable SF between photoactive pendants is unprecedented in the literature. The reversible and dynamic nature of supramolecular interactions allows to activate intermolecular SF at extremely dilute conditions (namely, 10 μM) by simply tailoring the experimental conditions (*i.e*., temperature and solvent polarity) that trigger the assembly.^50,51^ It is interesting to observe that the weak interchromophoric interactions between Pnc moieties, which play a key role in dictating the BTA-guided molecular arrangement within the assembly, are generally detrimental to fast and efficient singlet conversion in SF systems. Here, conformational dynamics likely modulate interactions between Pnc pendants, thus enabling SF in architectures with, on average, weakly coupling.

**Figure 4:**
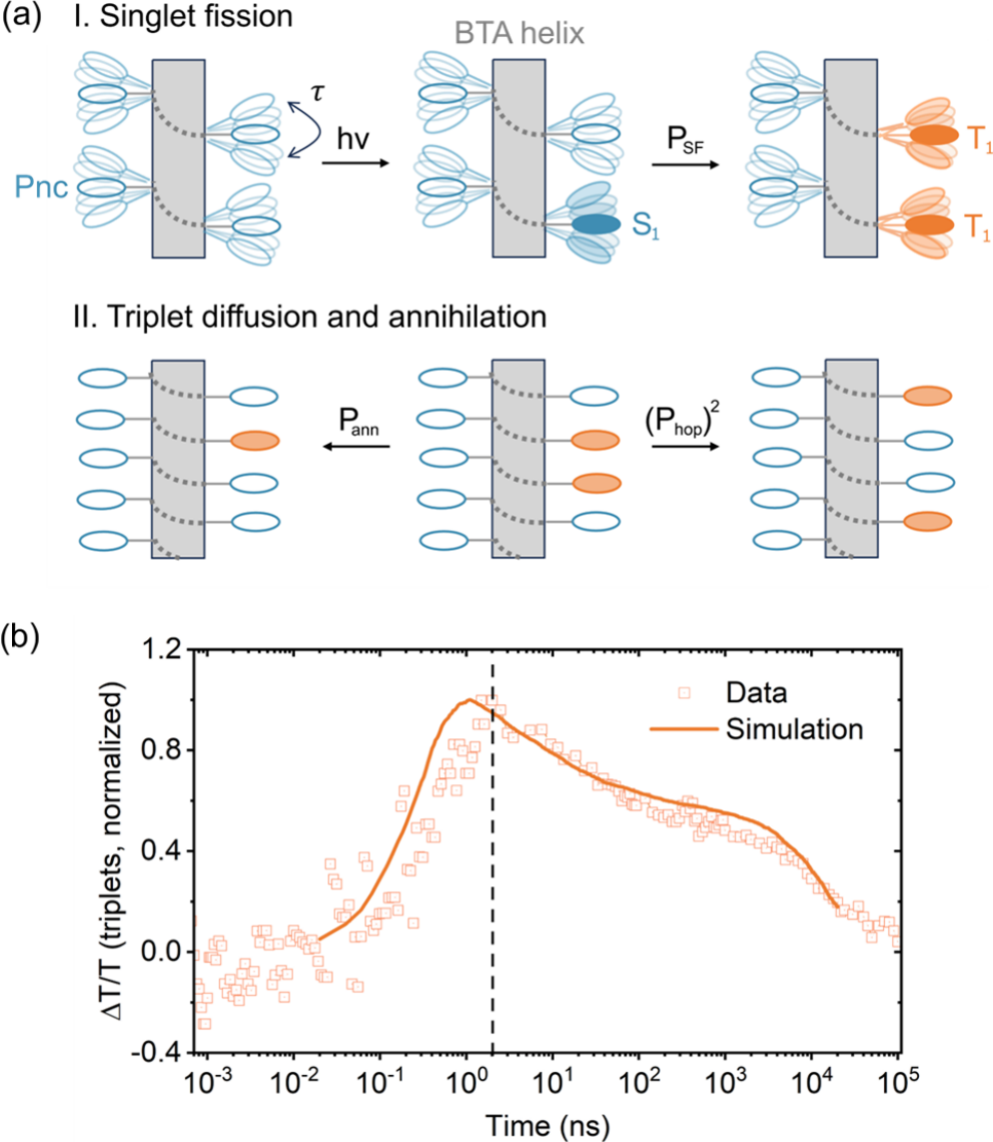
Modeling of triplet dynamics within the fibers. (a) Schematic representation of the SF process in BTA-Pnc supramolecular polymers. τ represents the frequency at which Pnc pendants interact with each other. *P*_SF_, *P*_hop_ and *P*_ann_ are the probabilities that a collision results in triplet formation, triplet hopping and triplet annihilation, respectively. (b) Comparison between measured (squares) and simulated (line) triplet kinetics in ps- and ns-regimes of 10 μM ***S*****-BTA-Pnc** in MCH at room temperature.

While global analysis validates that SF is operative in ***S*****-BTA-Pnc** supramolecular polymers, it does not allow to capture the dynamics of triplet generation and subsequent evolution within the fibers. To gain further insight into the role of time-dependent inter-Pnc interaction on the observed TA kinetics, we performed a full Monte Carlo simulation of triplet formation, diffusion and annihilation within the fibers (Figure 4, more details in SI, section 4.5). The triplet dynamics are regulated by the frequency at which two Pnc pendants interact, denoted by τ. Considering the probability of SF at the interaction of a Pnc in the singlet excited state with a ground-state Pnc as *P*_SF_, the probability for two Pnc triplet excitons on adjacent sites (*T*_1_··*T*_1_) to annihilate upon interaction as *P*_ann_, and the probability for triplet excitons to hop to an adjacent monomer in the ground state as *P*_hop_, the photophysical pathways for triplet formation and evolution can be described by the following equations:
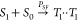
1
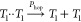
2
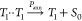
3
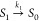
4
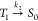
5Here, while eqs 1–3 describe pathways involving bimolecular interactions controlled by τ, eqs 4–5 denote the unimolecular decay of Pnc singlet and triplet excited states to the ground state (*k*_1_ = (11 ns)^−1^ and *k*_2_ = (15 μs)^−1^). As shown in Figure 4b, the simulation accurately reproduces the observed triplet kinetics for values of τ, *P*_SF_, *P*_hop_ and *P*_ann_ of 0.05 ps^–1^, 0.1, 0.075 and 0.0075, respectively (see section 4.5 in SI for details).

The results of the simulation reveal a key aspect of the excited state dynamics within the fibers. The value of *P*_ann_ is an order of magnitude smaller than *P*_hop_, suggesting that triplets in the supramolecular polymers are predisposed toward separation. The annihilation events in the fibers occur as free triplets hopping from site to site meet each other stochastically and is predominantly geminate. The geminate nature of annihilation is confirmed experimentally by following the triplet kinetics at different excitation fluences (Figure S23a).^52^ We speculate that the chiral organization within the Pnc framework–guided by the one-handed arrangement of amide-functionalized monomers–may have a role in regulating the annihilation kinetics. In other words, the combination of electric and magnetic transition dipole moments, possible only in chiral architectures, may predispose the triplets to separation. Intriguingly, a possible correlation between unusually long-lived photocharges and helical arrangement within H-type self-assembled nanowires has been proposed by Frauenrath and co-workers.^53^ In this connection, we anticipate that the role of helical chirality in disfavoring triplet–triplet annihilation kinetics will be an interesting direction for future work.

On the other hand, SF emerges as a useful tool to shed light on dynamics within self-assembled systems. As such, the frequency of interactions between Pnc pendants provides quantitative insights into the time-dependent fluctuations of molecular conformations within ***S*****-BTA-Pnc** supramolecular polymers. While the significant impact of dynamics on emergent properties in supramolecular systems is widely recognized in the literature, the characterization of conformational motions remains challenging due to the difficulty of adapting conventional experimental techniques to noncovalent assemblies.^54^

**Figure 5:**
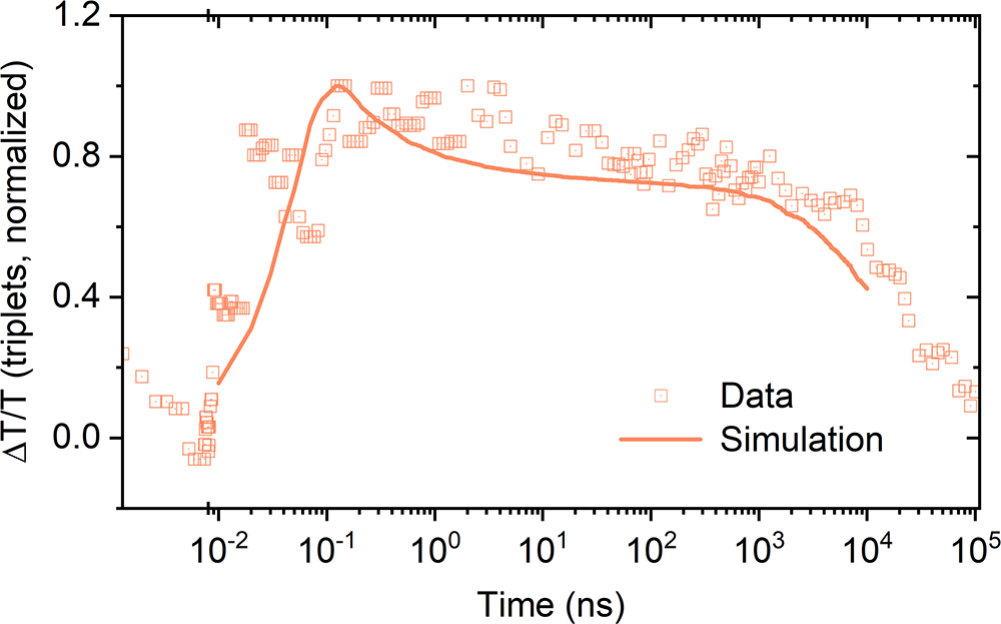
Triplet dynamics in **a-BTA-Pnc** supramolecular polymers. Measured (squares) and simulated (line) triplet kinetics in ps- and ns-regimes of 10 μM **a-BTA-Pnc** in MCH at room temperature.

To investigate how subtle changes in monomer design affect the excited state dynamics within the fibers, we examined the TA features of solutions of **a-BTA-Pnc** bearing linear (instead of branched) alkyl chains (Figure 1a). Although the spectral evolution for **a-BTA-Pnc** supramolecular polymers in solution is similar to that of ***S*****-BTA-Pnc** fibers, a closer look reveals a significant influence of the nature of the side chains on the triplet kinetics (Figures 5, S21b and S22). Global fitting of the ps-TA data reveals that SF is significantly faster (about 1.75 times) in **a-BTA-Pnc** fibers compared to ***S*****-BTA-Pnc** fibers (Figure S24, see section 4.4 in SI for further details). On the other hand, simulation of triplet kinetics can reproduce the overall dynamics in **a-BTA-Pnc** fibers for values of τ, *P*_SF_, *P*_hop_ and *P*_ann_ of 0.1 ps^–1^, 0.4, 0.3 and 0.09, respectively (Figure 5). The values of τ and *P*_SF_ in **a-BTA-Pnc** fibers are higher than those found for ***S*****-BTA-Pnc** fibers, which explains the faster rate of SF in the former. This translates into a higher triplet generation yield in **a-BTA-Pnc** fibers (Table S2). Similarly to ***S*****-BTA-Pnc** fibers, *P*_hop_ is significantly higher than *P*_ann_, indicating that triplets are predisposed to separation.^55^ In this regard, it should be noted that even for **a-BTA-Pnc**, unfavorable annihilation kinetics could result from the chiral arrangement of monomers within enantiopure fibers. In fact, racemic mixtures of one-handed helical assemblies are formed upon supramolecular polymerization of achiral BTA discotics.^34^ The overall triplet kinetics are largely controlled by *P*_ann_ (Figure S29), but even with significant values of *P*_ann_, the simulations suggest that the triplet yield in **a-BTA-Pnc** fibers exceeds 100% for at least 100 ns (see section 4.5 in SI for further details).

Overall, these results indicate that the molecular arrangement within the assembly plays a critical role in regulating interchromophoric interactions between photoactive pendants.^56^ Namely, the higher values of τ, *P*_SF_, *P*_hop_ and *P*_ann_ in **a-BTA-Pnc** fibers compared to the branched counterpart are consistent with the tighter packing of BTA cores within the supramolecular backbone as evidenced by chiroptical studies (*vide supra*). In other words, the molecular arrangement within **a-BTA-Pnc** fibers is tuned to maintain high triplet yields over long time scales as a result of fast and efficient triplet formation and hopping.

## Conclusions

Here, helical supramolecular polymers decorated with Pnc motifs emerge as a versatile tool to elucidate intermolecular SF dynamics in solution, enabling the formation of exceptionally long-lived mobile triplets while ensuring fast and efficient singlet conversion. By exploiting the hydrogen bond-driven assembly of BTA cores into one-dimensional scaffolds, we direct the organization of photoactive pendants into long-range helical domains. In this way, a strategy emerges to mitigate the strength of interchromophoric interactions while imparting crystalline-mimicking supramolecular order. Pump–probe measurements and global analysis of the TA data show that SF is the dominant mechanism for singlet decay in the supramolecular fibers, revealing fast singlet conversion within hundreds of picoseconds and TQY well above 100%. Moreover, a remarkably slow decay of triplet excitons is observed on the time scale of tens of microseconds. Monte Carlo simulations allow to unravel the kinetics of triplet generation and evolution within the supramolecular polymers, being regulated by dynamic interactions between weakly coupled photoactive pendants. Simulations reveal that triplet exciton diffusion along the fibers is favored over annihilation, resulting in mobile free triplets able to diffuse about 100 monomer units apart. Changes in the molecular packing within the one-dimensional assembly have a significant impact on the excited state kinetics, affording TQY exceeding 100% for at least 100 ns as a result of fast and efficient triplet formation and hopping.

These findings provide new insights into the quest for long-lived triplets in SF architectures, opening a new window for the exploitation of conformational dynamics in weakly coupled systems and raising crucial questions on the correlation between chiral organization and triplet–triplet recombination in supramolecular aggregates.

## Supporting Information Available

Experimental methods, synthetic procedures and characterization data, additional spectra and simulations (PDF)
