# Supplementary material for: Long-Lived Triplets from Singlet Fission in Pentacene-Decorated Helical Supramolecular Polymers
Source: J Am Chem Soc. 2024 Oct 8;146(42):28985–93. doi: 10.1021/jacs.4c09844 (PMC11505394; doi:10.1021/jacs.4c09844)
Supplement: Supplementary file 1 — ja4c09844_si_001.pdf [file ja4c09844_si_001.pdf]

Supporting Information

Long-Lived Triplets from Singlet Fission in Pentacene-Decorated Helical Supramolecular Polymers

Giulia Lavarda,[a] Ashish Sharma,[b] Marko Beslać,[a] Stef A. H. Jansen,[a] Stefan C. J. Meskers,[a] Richard H. Friend,[b] Akshay Rao,[b]* and E. W. Meijer [a]**

[a] Institute for Complex Molecular Systems and Laboratory of Macromolecular and Organic Chemistry, Eindhoven University of Technology, 5600 MB Eindhoven, The Netherlands.

[b] Department of Physics, Cavendish Laboratory, University of Cambridge, Cambridge CB30HE, United Kingdom.

* Correspondence to: g.lavarda@tue.nl, ar525@cam.ac.uk, e.w.meijer@tue.nl

Table of Contents

1. Materials and methods……S1
2. Synthesis and characterization……S4
3. Self-assembly studies……S10
  3.1 UV-vis absorption, circular dichroism and FT-IR spectroscopy……S10
  3.2 Computational analysis of circular dichroism data……S13
  3.3 Atomic force microscopy……S16
4. Photophysical characterization……S18
  4.1 Steady-state emission spectroscopy……S18
  4.2 Transient absorption spectroscopy……S18
  4.3 Triplet yield quantification……S21
  4.4 Global fits……S22
  4.5 Simulation of triplet dynamics in fibers……S24
5. Molecular modeling……S28
6. Author contributions……S44
7. References……S45

1. Materials and methods

(1-Cyano-2-ethoxy-2-oxoethylidenaminooxy)dimethylamino-morpholino-carbenium hexafluorophosphate (COMU) and *N*,*N*-diisopropylethylamine (DIPEA) were purchased from Sigma-Aldrich. Solvents used for synthesis were purchased from Biosolve B.V. Deuterated solvents were purchased from Cambridge Isotopes Laboratories. Spectroscopic grade methylcyclohexane (MCH) and chloroform were purchased from Thermo Fisher Scientific and Sigma-Aldrich, respectively, and dried over molecular sieves before use. All other chemicals and solvents were used as received. The synthesis of Pnc-NH_2 will be reported in due course. The synthesis of *N*,*N'*,*N''*-tris-(*n*-octyl)benzene-1,3,5-tricarboxamide (a-BTA),[1] *N*,*N'*,*N''*-tris((*S*)-3,7-dimethyloctyl)benzene-1,3,5-tricarboxamide (*S*-BTA),[1] 3,5-bis-*n*-octylaminocarbonyl-benzoic acid (a-BTA-COOH),[2] and 3,5-bis(*S*)-3,7-dimethyloctylaminocarbonyl-benzoic acid (*S*-BTA-COOH)[3] has been reported elsewhere.

Thin layer chromatography (TLC) was carried out on aluminum sheets precoated with silica gel 60 F254 (Merck). Size exclusion chromatography was performed on Bio-BeadsTM S-X1 Support (200-400 mesh). ^{1}H and ^{13}C nuclear magnetic resonance (NMR) spectra were recorded on a Bruker ASCEND spectrometer. In ^{1}H NMR and ^{13}C NMR spectra, chemical shifts (δ) are expressed in ppm and referenced to the residual peak of the solvent. Matrix-assisted laser desorption/ionization time of flight (MALDI-TOF) mass spectrometry (MS) was performed with a Bruker Autoflex Speed instrument. *Trans*-2-[3-(4-*tert*-butylphenyl)-2-methyl-2-propenylidene] malononitrile (DCTB) and cyano-4-hydroxycinnamic acid (CHCA) were used as matrices.

For all spectroscopic measurements, sealed Hellma and Spectrocell quartz cuvettes were used. Samples for spectroscopic measurements were prepared by weighing the desired amount of compound, transferring it to a sealed vial and adding the required volume of solvent (either MCH or chloroform) using Gilson MICROMAN positive-displacement pipettes to reach the target concentration. The solutions were then sonicated in a 40 °C water bath for 60 seconds and vortexed for 30 seconds. They were then transferred to sealed cuvettes and degassed with Ar. For the preparation of multicomponent solutions, solutions of the individual components were first prepared in separate vials following the procedure described above. The required volumes were then transferred to a sealed cuvette and the resulting solution was degassed with Ar. All spectroscopic measurements were performed on freshly prepared solutions.

Steady-state UV-vis absorption, circular dichroism (CD) and fluorescence spectroscopy were performed on a JASCO J-815 CD spectrometer equipped with a JASCO MPTC-490S temperature controller and a JASCO FMO-427S/15 emission monochromator. Variable temperature (VT) measurements and single measurements at 80 °C were performed after heating the sample to 80 °C for 10 minutes in the spectrophotometer. Single measurements at 20 °C were performed after heating the sample to either 80 °C (MCH) or 50 °C (chloroform) for 10 minutes and then cooling to 20 °C at a rate of 1 °C/min. A cooling rate of 1 °C/min was used for VT measurements. For full spectra measurements at VT, the cooling ramp was stopped during the acquisition of the spectra.

Time-correlated single photon counting (TCSPC) measurements were performed on an Edinburgh Instruments LifeSpec-PS spectrometer equipped with a temperature controller. TCSPC measurements at 80 °C were performed after heating the sample to 80 °C for 10 minutes in the instrument. After controlled cooling at a rate of 1 °C/min, TCSPC data at 20 °C were collected. TCSPC data were analyzed using the Levenberg-Marquardt algorithm for non-linear least squares fitting.

Transient absorption (TA) measurements in the picosecond (ps) and nanosecond (ns) time-domains were performed using custom setups. For ps-TA measurements, the light source was a Light Conversion PHAROS, which provides 1030 nm pulses (400 μJ per pulse) with a repetition rate of 38 kHz. The pump beam (660 nm, 250 fs full width half maximum) is generated using a narrow-band optical parametric oscillator system (ORPHEUS-LYRA, Light conversion) with 1030 nm seed. On-off pump pulses are generated by means of a mechanical chopper (Thorlabs) before incidence on the sample. The white light probe beam (520 -900 nm) is generated using a 4 mm YAG substrate and the probe pulse is delayed relative to the pump pulse using a computer-controlled mechanical delay-stage (Newport). The pump and probe beams are overlapped on the sample and then the probe transmitted through the sample collected using a silicon line scan camera (AViiVA EM2/EM4 with a visible monochromator with 550 nm blazed grating). For ns-TA, the pump is generated by the third harmonic (355 nm) of a Q-switched Nd:YVO4 (1 ns pump length, Advanced Optical Technologies Ltd AOT-YVO-25QSPX). The probe beam was generated with a LEUKOS Disco 1 UV supercontinuum laser (STM-1-UV, 1 kHz) and delayed electronically with respect to the pump. To measure the TA response, the pump and probe beams are overlapped on the sample and focused into an imaging spectrometer (Andor, Shamrock SR 303i). The beams are detected using a pair of linear image sensors (Hamamatsu, G11608) driven and read out at the full laser repetition rate by a custom-built board from Stresing Entwicklungsburo. A chopper (500 Hz) is used to generate on-off pump pulses to calculate differential transmission, $\Delta T/T$. TA measurements were performed at room temperature after heating the sample to either 80 °C (MCH) or 50 °C (chloroform) for 10 minutes and then cooling at a rate of 1 °C/min. Room temperature solutions were then degassed with nitrogen.

Fourier transform-infrared (FT-IR) spectra were acquired using a Perkin Elmer Spectrum Two spectrometer equipped with a liquid cell with CaF_2 windows and a slide holder module.

Atomic force microscopy (AFM) images were collected using a Cypher Environmental Scanner (ES) equipped with a closed cell and a normal laser diode. A heating and cooling stage was used to actively control the temperature at 20 °C. Silicon NCSTR probes (Oxford Instruments, spring constant k = 7.4 N/m; f = 160 kHz) with a tip height of 10-15 μm and a radius of 7 nm were used for all measurements. The AFM tip was thermally calibrated using the 'Get Real' function of the Igor Pro software. Subsequent height images were acquired in repulsive tapping mode (phase <90) at a resolution of 1024x1024 pixels using a scan rate of ~2 Hz and an integral gain of 50-150. Image contrast was enhanced using first order planefit and flattening with Gwyddion v2.60. Samples were prepared by either spin-coating or drop-casting 10 μM solutions of the compound in MCH onto freshly cleaved 1.5x1.5 cm^2 sized mica. Prior to deposition, the solutions were heated in the spectrophotometer to 80 °C for 10 minutes and then cooled to 20 °C at a rate of 1 °C/min. A speed of 1000 rpm was used for the preparation of spin-coated samples.

The thermodynamic mass-balance model for cooperative supramolecular polymerization is derived in Section 3.2. Fitting was optimized in Matlab® version R2020a.

Computational analysis of the BTA-Pnc hexamer was performed with Schrödinger Maestro Suite 2020-4. The input structure was generated using the built-in 2D structure generator followed by geometry minimization with Macromodel (OPLS4 force field, vacuum). The hexamer structure was oriented such that linear hydrogen bonds along the helical screw axes are formed and then minimized with Macromodel.

2. Synthesis and characterization

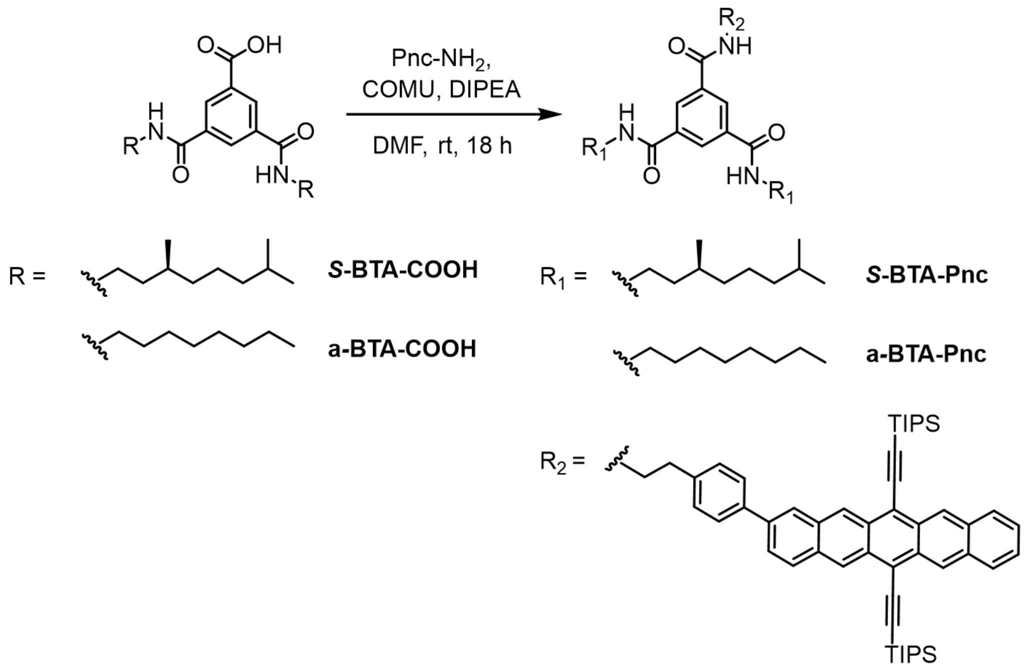

Scheme S1. Synthesis of ***S*-BTA-Pnc** and **a-BTA-Pnc** from the corresponding ***S*-BTA-COOH** and **a-BTA-COOH** precursors.

<u>Synthesis of ***S*-BTA-Pnc**</u>

3,5-bis-(*S*)-3,7-dimethyloctylaminocarbonyl-benzoic acid (50.0 mg, 102 µmol), COMU (54.8 mg, 128 µmol) and DIPEA (32.8 µL, 188 µmol) were dissolved in DMF (2 mL) in a 10 mL round bottom flask and the reaction mixture was stirred at room temperature for 15 min. At this point, Pnc-NH_2 (97.0 mg, 128 µmol) was added, and the mixture was stirred overnight at room temperature. To limit light exposure, the reaction flask was covered with aluminum foil. Then, the crude product was dissolved in ethyl acetate (15 mL). The solution was washed with aqueous HCl (1 M, 3x15 mL), saturated $NaHCO_3$ (3x15 mL) and brine (2x15 mL). The organic phase was dried over $MgSO_4$, filtered, and evaporated to dryness under reduced pressure. The crude was purified by size exclusion chromatography using chloroform as eluent. Recrystallization from hexane afforded ***S*-BTA-Pnc** as a blue solid in 41% yield (51.0 mg).

^1H NMR (400 MHz, Chloroform-d_1): δ (ppm) = 9.32-9.30 (m, 4H), 8.33 (s, 3H), 8.12 (s, 1H), 8.04 (d, J = 9.0 Hz, 1H), 7.98-7.96 (m, 2H), 7.76 (d, J = 8.2 Hz, 2H), 7.70 (dd, J_1= 9.0 Hz, J_1= 1.8 Hz, 1H), 7.43–7.39 (m, 4H), 6.62 (t, J = 5.9 Hz, 1H), 6.40 (t, J = 5.6 Hz, 2H), 3.85-3.80 (m, 2H), 3.54-3.42 (m, 4H), 3.04 (t, J = 7.3 Hz, 2H), 1.68–1.60 (m, 4H), 1.54–1.49 (m, 4H), 1.44–1.37 (m, 42H), 1.33–1.26 (m, 6H), 1.18–1.10 (m, 6H), 0.93 (d, J = 6.5 Hz, 6H), 0.85 (d, J = 6.6 Hz, 12H).
^{13}C NMR (101 MHz, Chloroform-d_1) : δ (ppm) = 165.99, 165.71, 139.40, 138.22, 139.01, 135.50, 135.26, 132.56, 132.47, 132.42, 131.60, 131.08, 130.88, 130.75, 129.52, 128.82, 128.20, 128.08, 127.74, 126.66, 126.47, 126.27, 126.18, 125.81, 118.60, 118.40, 107.41, 107.30, 104.84, 104.81, 41.70, 39.37, 38.69, 37.25, 36.76, 35.60, 30.90, 28.08, 24.76, 22.83, 22.73, 19.62, 19.17, 19.15, 11.83.
MS (MALDI-TOF): m/z calculated for $C_{81}H_{109}N_3O_3Si_2$: 1227.80; found: 1227.78 [M^+].

Synthesis of **a-BTA-Pnc**

3,5-bis-*n*-octylaminocarbonyl-benzoic acid (40.0 mg, 92 μmol), COMU (49.5 mg, 116 μmol) and DIPEA (28.7 μL, 165 μmol) were dissolved in DMF (2 mL) in a 10 mL round bottom flask and the reaction mixture was stirred at room temperature for 15 min. At this point, Pnc-NH_2 (87.6 mg, 116 μmol) was added, and the mixture was stirred overnight at room temperature. To limit light exposure, the reaction flask was covered with aluminum foil. Then, the crude product was dissolved in ethyl acetate (15 mL). The solution was washed with aqueous HCl (1 M, 3x15 mL), saturated $NaHCO_3$ (3x15 mL) and brine (2x15 mL). The organic phase was dried over $MgSO_4$, filtered, and evaporated to dryness under reduced pressure. The crude was purified by size exclusion chromatography using chloroform as eluent. Recrystallization from hexane afforded **a-BTA-Pnc** as a blue solid in 42% yield (45.1 mg).

^{1}H NMR (400 MHz, Chloroform-d_1): δ (ppm) = 9.32-9.30 (m, 4H), 8.33 (m, 3H), 8.11 (s, 1H), 8.04 (d, J = 9.2 Hz, 1H), 7.98-7.96 (m, 2H), 7.76 (d, J = 8.2 Hz, 2H), 7.69 (dd, J_1= 8.9 Hz, J_1= 1.8 Hz, 1H), 7.42–7.39 (m, 4H), 6.72 (t, J = 4.9 Hz, 1H), 6.52 (t, J = 5.6 Hz, 2H), 3.84-3.79 (m, 2H), 3.47-3.42 (m, 4H), 3.04 (t, J = 7.2 Hz, 2H), 1.68–1.58 (m, 4H), 1.41–1.37 (m, 42H), 1.32–1.25 (m, 20H), 0.86 (t, J = 7.1 Hz, 6H).
^{13}C NMR (101 MHz, Chloroform-d_1) : δ (ppm) = 166.05, 165.79, 139.37, 138.23, 137.98, 135.52, 135.29, 132.55, 132.46, 132.42, 131.59, 131.07, 130.88, 130.75, 129.52, 128.81, 128.23, 128.09, 127.72, 126.65, 126.47, 126.27, 126.18, 125.78, 118.59, 118.39, 107.40, 107.30, 104.84, 104.80, 41.68, 40.57, 35.58, 31.93, 29.70, 29.41, 29.33, 27.15, 22.77, 19.17, 19.15, 14.21, 11.83.
MS (MALDI-TOF): m/z calculated for $C_{77}H_{101}N_3O_3Si_2$: 1171.74; found: 1171.72 [M^+].

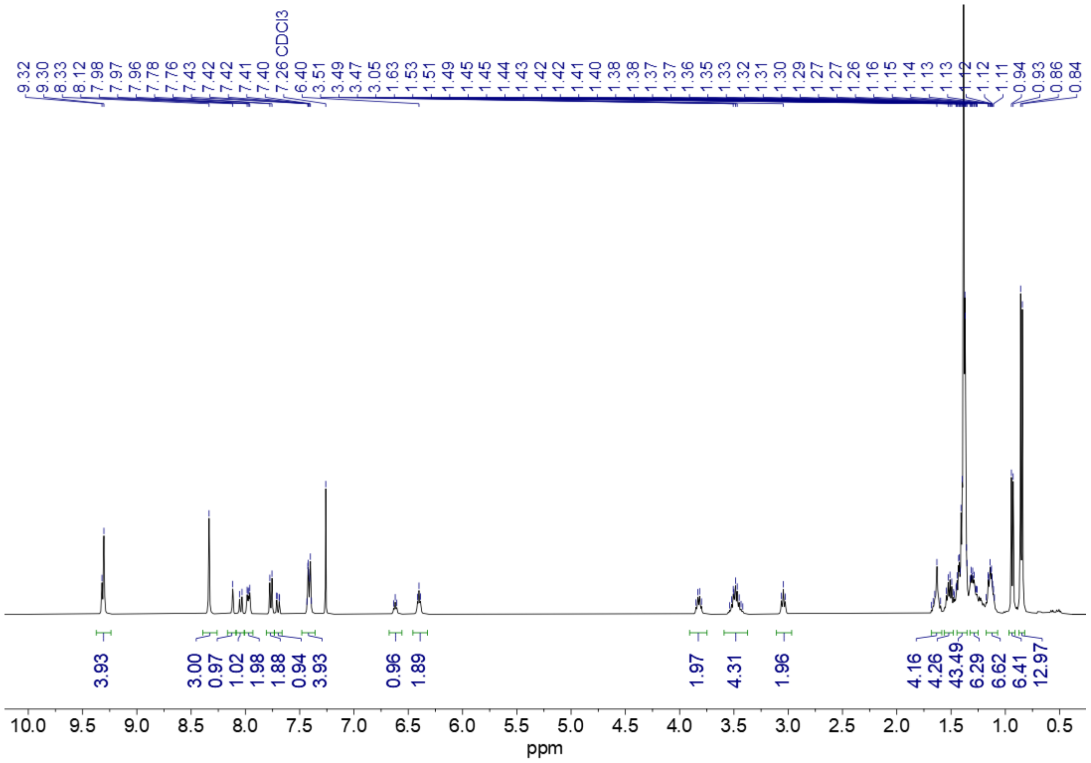

Figure S1. ^{1}H NMR spectrum (400 MHz, chloroform-d_1) of ***S*-BTA-Pnc**.

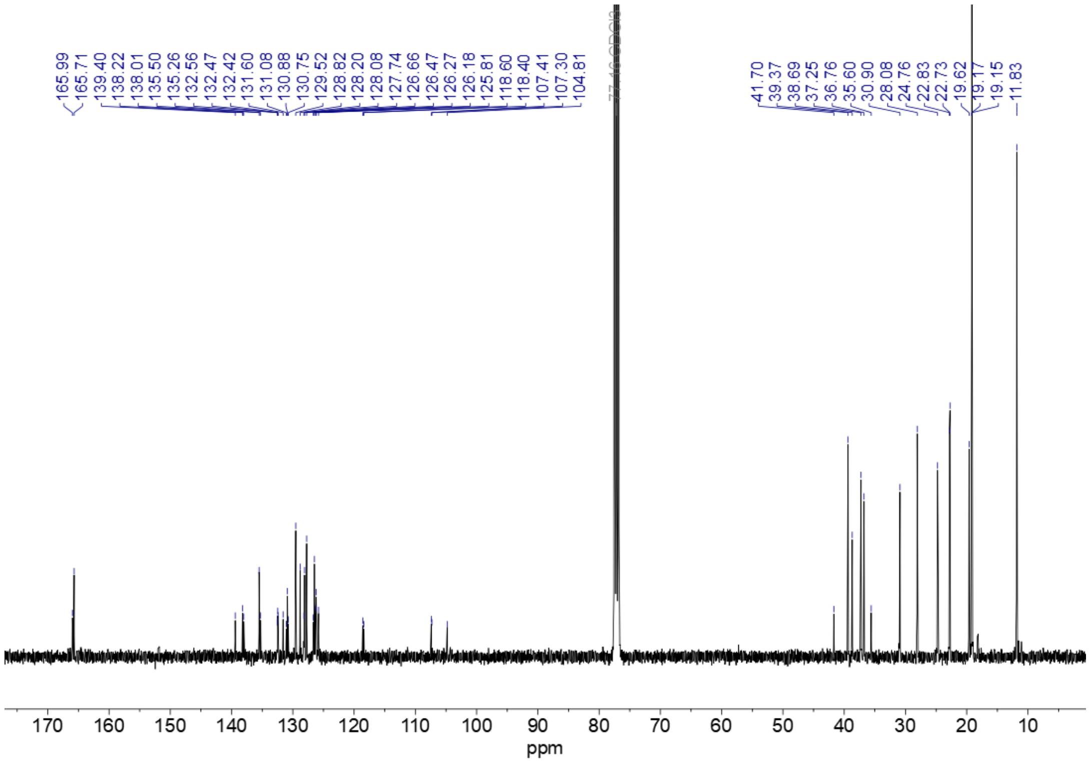

Figure S2. ^{13}C NMR spectrum (101 MHz, chloroform-d_1) of ***S*-BTA-Pnc**.

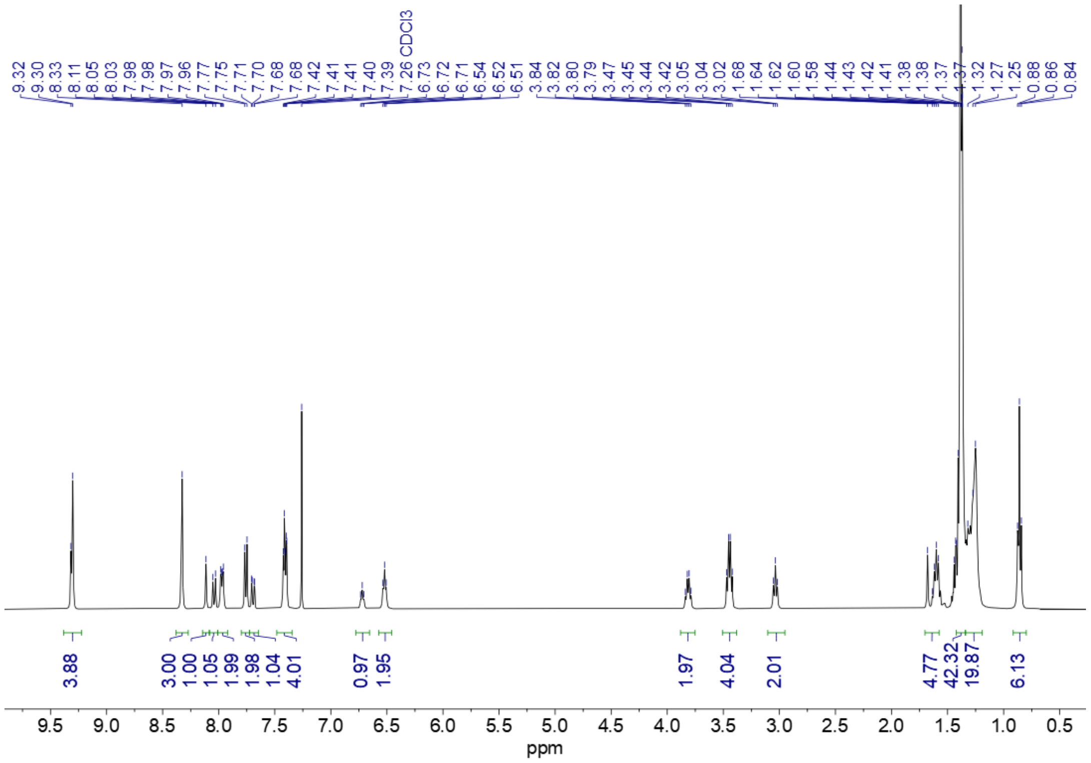

Figure S3. ^{1}H NMR spectrum (400 MHz, chloroform-d_1) of **a-BTA-Pnc**.

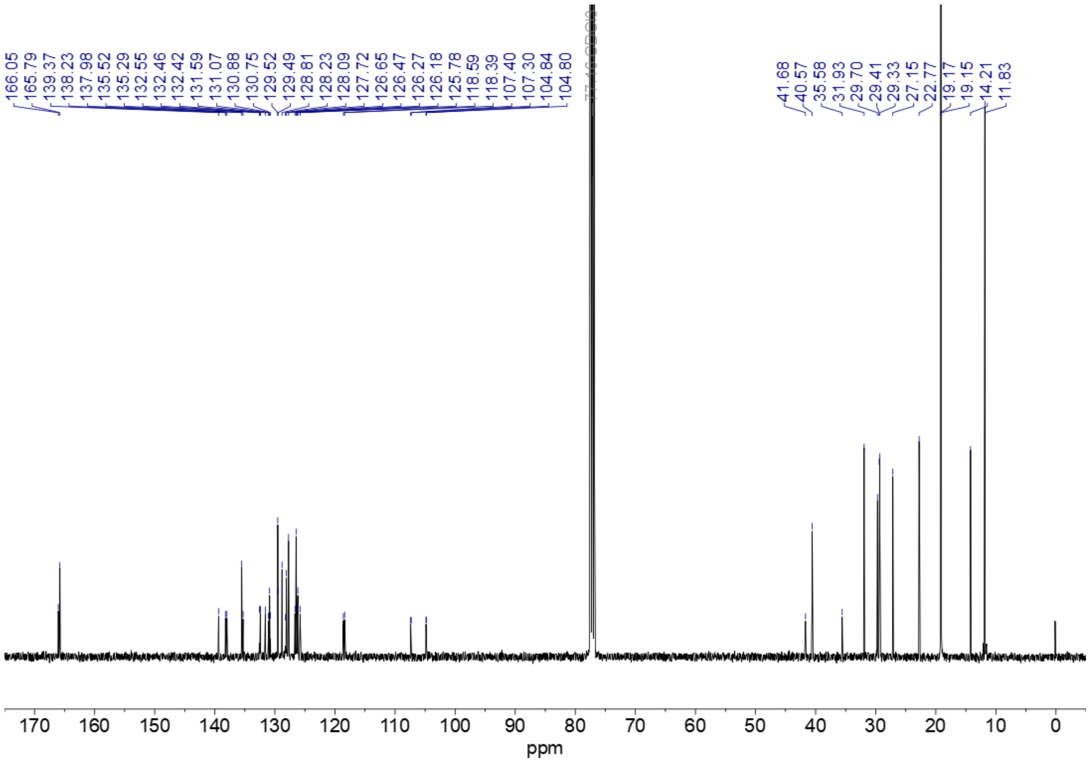

Figure S4. ^{13}C NMR spectrum (101 MHz, chloroform-d_1) of **a-BTA-Pnc**.

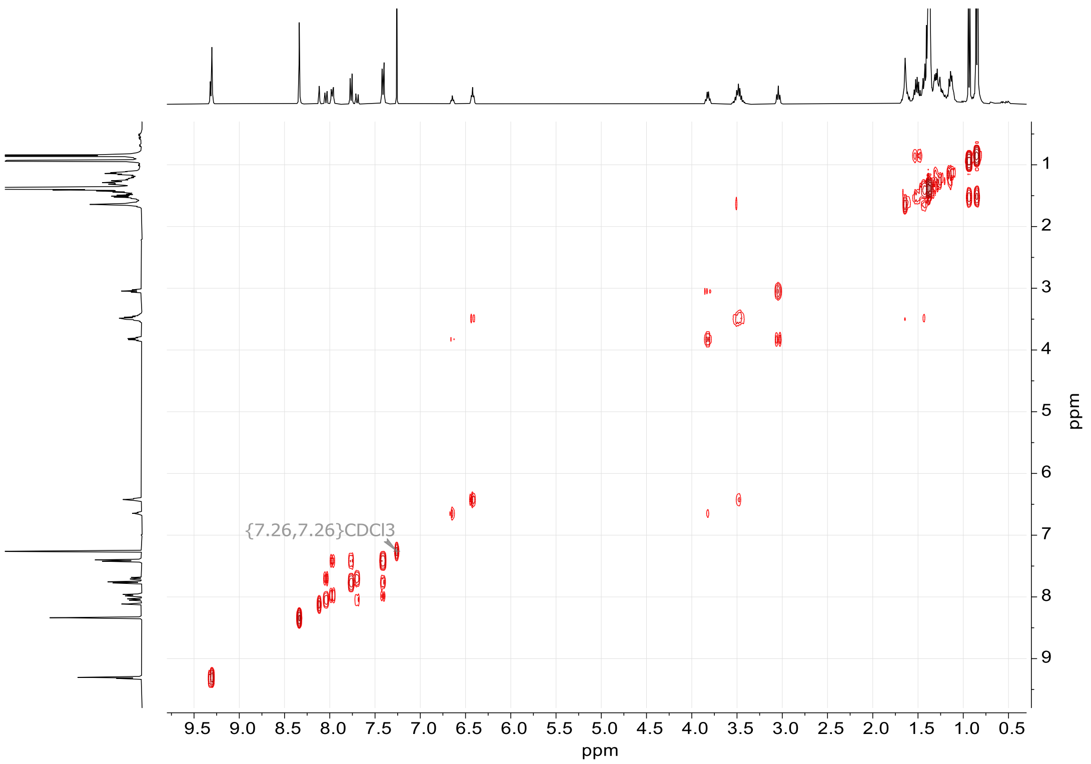

Figure S5. H-H COSY NMR spectrum (400 MHz, chloroform-d_1) of ***S*-BTA-Pnc**.

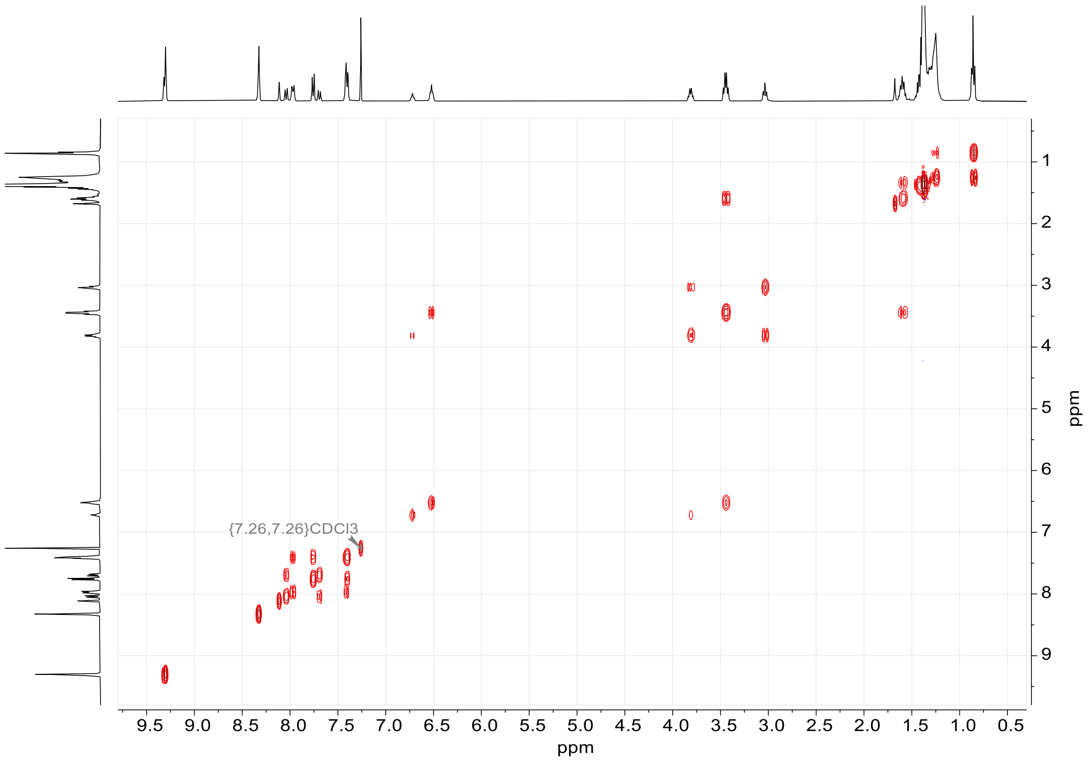

Figure S6. H-H COSY NMR spectrum (400 MHz, chloroform-d_1) of **a-BTA-Pnc**.

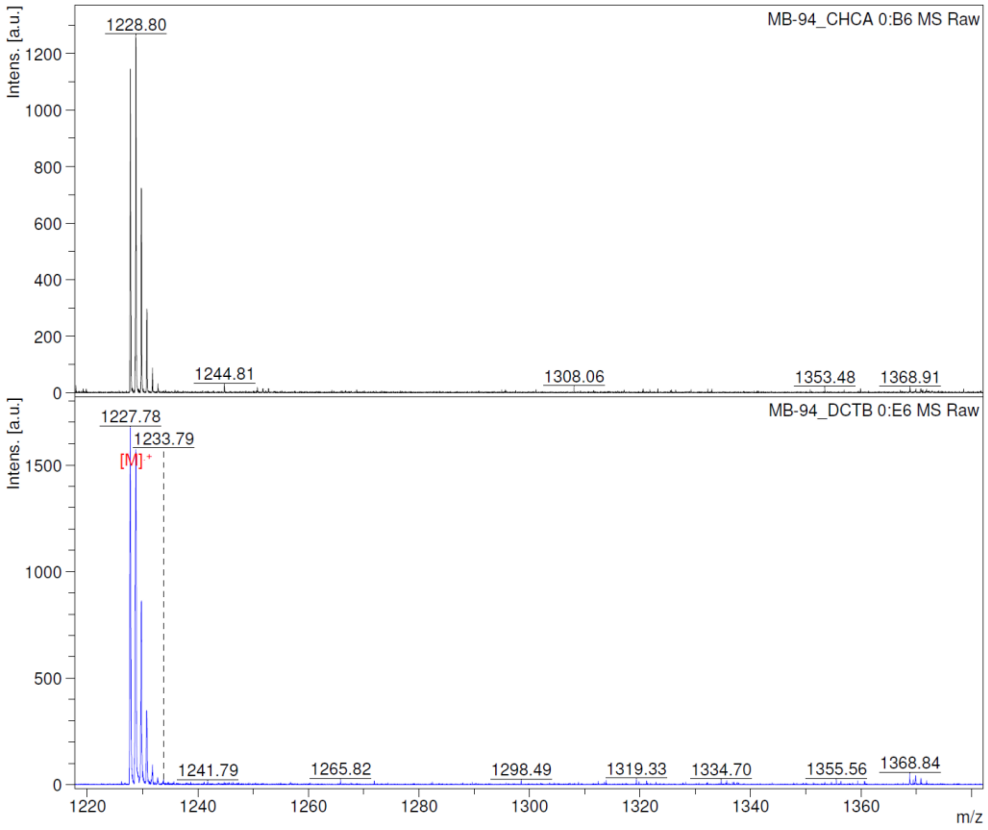

Figure S7. MALDI-TOF mass spectrum (in CHCA and DCTB matrices) of ***S*-BTA-Pnc**.

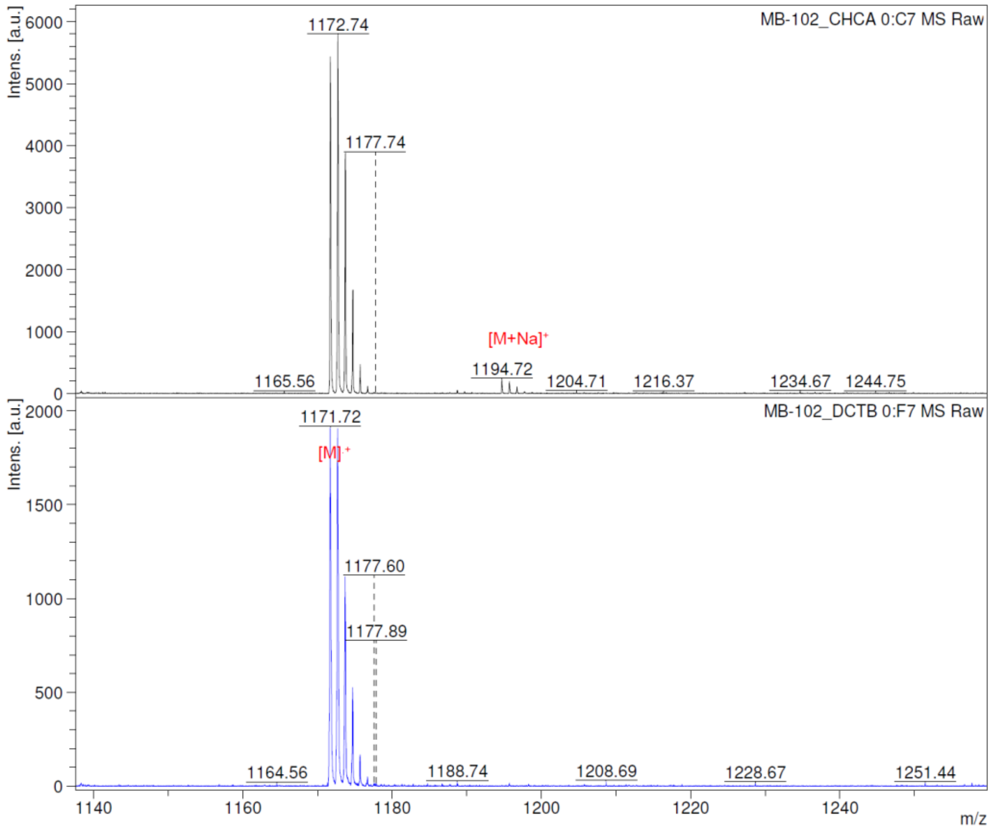

Figure S8. MALDI-TOF mass spectrum (in CHCA and DCTB matrices) of **a-BTA-Pnc**.

3. Self-assembly studies

3.1 UV-vis absorption, circular dichroism and FT-IR spectroscopy

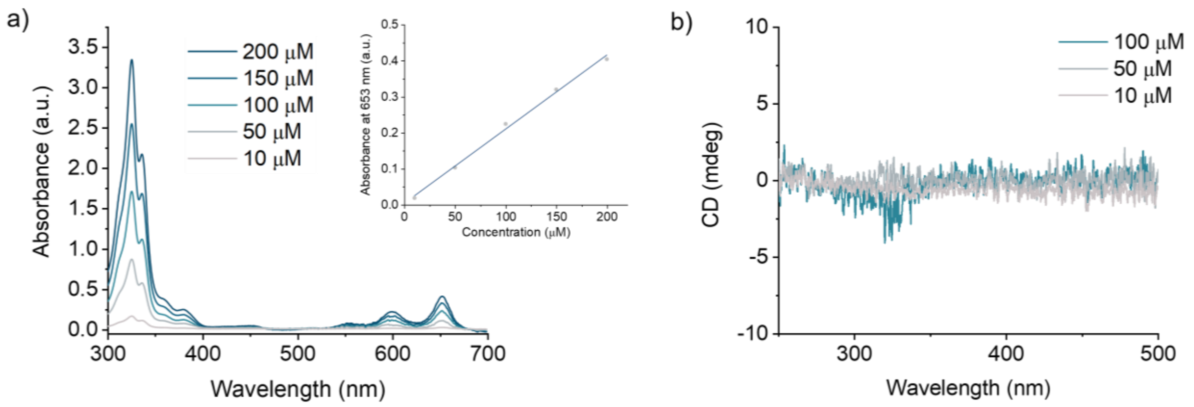

Figure S9. Concentration-dependent a) UV-vis absorption and b) CD spectra of ***S*-BTA-Pnc** solutions in chloroform at 20 °C. Measurements were performed after heating the samples to 50 °C, equilibrating at 50 °C for 10 minutes and then cooling to 20 °C at a rate of 1 °C/min. Optical path length: 1 mm. Inset in a) Linear correlation between absorbance at 653 nm and concentration. No evidence of assembly is observed.

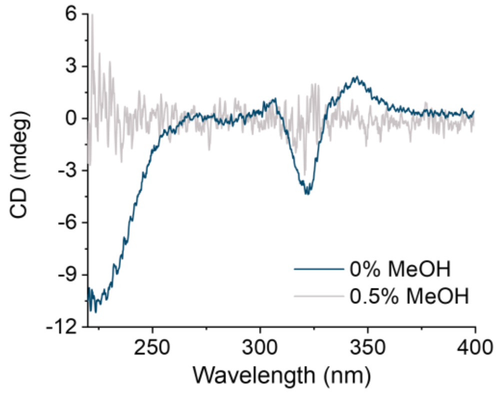

Figure S10. CD spectra of 10 μM ***S*-BTA-Pnc** in MCH at 20 °C before (blue line) and after (gray line) the addition of MeOH as a hydrogen bond scavenger. MeOH was added after heating the sample to 80 °C, equilibrating at 80 °C for 10 minutes and then cooling to 20 °C at a rate of 1 °C/min. Optical path length: 10 mm. The disappearance of the CD signal attributable to ***S*-BTA-Pnc** supramolecular polymers upon addition of MeOH indicates the rupture of the helical aggregates.

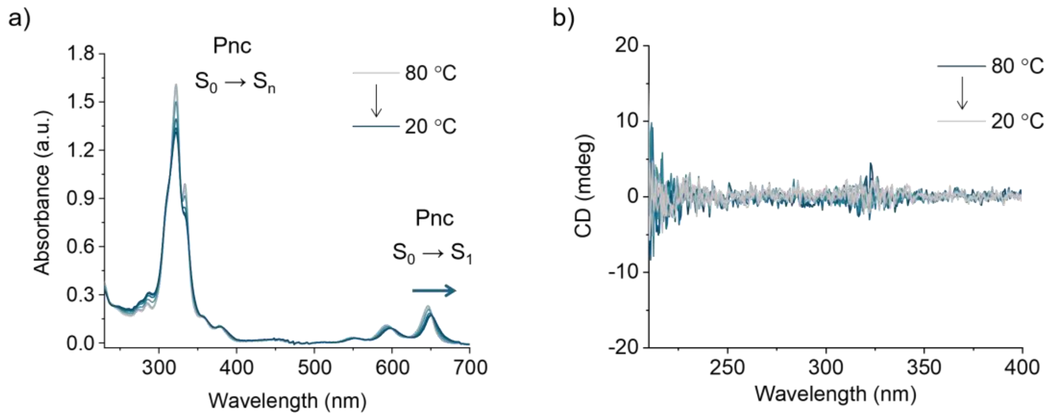

Figure S11. a) VT-Absorption and b) VT-CD spectra of 10 μM **a-BTA-Pnc** in MCH measured upon controlled cooling from 80 °C to 20 °C at a rate of 1 °C/min (optical path length: 10 mm). The spectral changes observed upon cooling – including a small bathochromic shift (namely 5 nm) of the low-energy Pnc absorption – are similar to that of the branched ***S*-BTA-Pnc** analog (Figure 2a), indicating a comparable molecular arrangement of the pentacene pendants within the aggregates. The absence of CD signal is due to the formation of equal amounts of *M* and *P* helices.

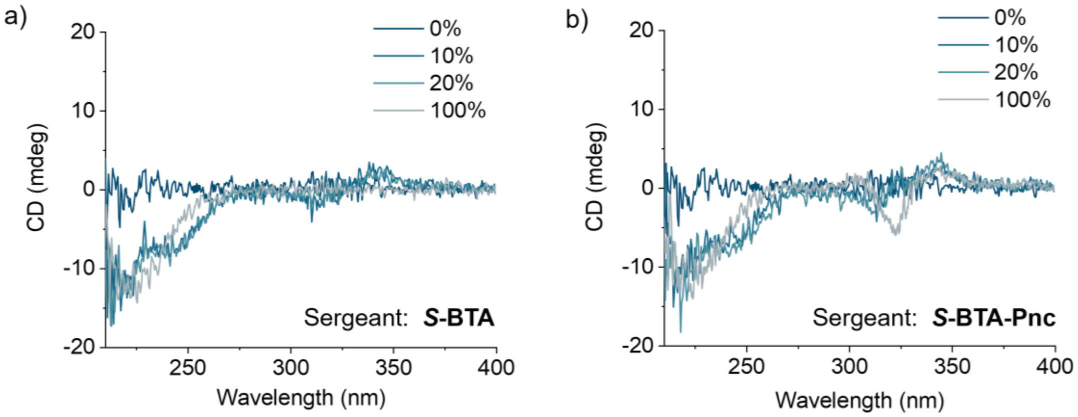

Figure S12. CD spectra of 10 μM a) **a-BTA-Pnc/*S*-BTA** mixtures and b) **a-BTA-Pnc/*S*-BTA-Pnc** mixtures in MCH at 20 °C in the presence of different molar ratios of chiral sergeant (optical path length: 10 mm). No CD signal is observed for **a-BTA-Pnc** homopolymers (0% molar ratio of chiral sergeant) due to the formation of equal amounts of *M* and *P* helices. The presence of non-linear CD signals for mixtures containing ***S*-BTA** or ***S*-BTA-Pnc** indicates that the chiral sergeant copolymerize with **a-BTA-Pnc** soldiers and dictate the helicity of the copolymers. The overlap between CD spectra obtained in the presence of 10% or 20% molar ratios of chiral sergeant indicates that the former amount is sufficient to fully bias the helicity of copolymers. The difference in shape between the CD profile of **a-BTA-Pnc/*S*-BTA** copolymers and the CD profile of ***S*-BTA** homopolymers and between the CD profile of **a-BTA-Pnc/*S*-BTA-Pnc** copolymers and the CD profile of ***S*-BTA-Pnc** homopolymers is due to the different packing of **a-BTA-Pnc** and ***S*-BTA-Pnc** (or ***S*-BTA**) within the supramolecular stacks.

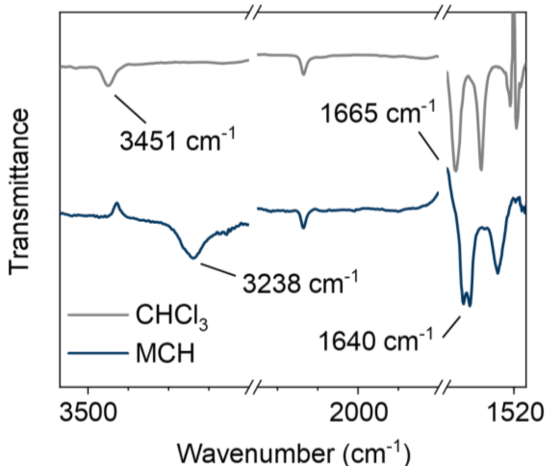

Figure S13. FT-IR spectra of 0.5 mM **a-BTA-Pnc** in chloroform and MCH.

3.2 Computational analysis of circular dichroism data

The supramolecular polymerization is modeled using thermodynamic mass-balance expressions.[4] In the model, the polymers (P) are assumed to grow by monomer (M) addition and dissociation at the chain ends. The reactions that describe the cooperative pathway, for which a nucleus size of 2 is assumed, are:

$$\begin{aligned}
&\mathrm{M}+\mathrm{M} \underset{}{\overset{K_\mathrm{n}}{\rightleftharpoons}} \mathrm{P}_2\\
&\mathrm{P}_2+\mathrm{M} \underset{}{\overset{K_\mathrm{e}}{\rightleftharpoons}} \mathrm{P}_3\\
&\mathrm{P}_3+\mathrm{M} \underset{}{\overset{K_\mathrm{e}}{\rightleftharpoons}} \mathrm{P}_4\\
&\quad\vdots\\
&\mathrm{P}_i+\mathrm{M} \underset{}{\overset{K_\mathrm{e}}{\rightleftharpoons}} \mathrm{P}_{i+1}\\
&\quad\vdots
\end{aligned} \tag{S1}$$

where K_n is the nucleation constant and K_e is the elongation constant of the nucleated pathway. Assuming that the activity of the chemical species is equal to their concentrations, the concentration of monomers in *i*-mer in the cooperative aggregates at thermodynamic equilibrium can then be expressed as a function of the free monomer concentration with:

$$[\mathrm{P}_i] = i\cdot\sigma\cdot K_\mathrm{e}^{i-1}\cdot[\mathrm{M}]^i \text{ for } i \geq 2 \tag{S2}$$

where [M] is the equilibrium monomer concentration and σ is the cooperativity parameter, which is $\sigma=K_\mathrm{n}/K_\mathrm{e}$.

The total concentration of M in the system is the sum of monomers and nucleated aggregates:

$$\begin{aligned}
[\mathrm{M}]_\mathrm{tot} &= [\mathrm{P}]_\mathrm{tot} + [\mathrm{M}]\\
&= \left(\sum_{i=2}^{\infty}[\mathrm{P}_i]\right)+[\mathrm{M}]\\
&= \left(\sum_{i=1}^{\infty} i\cdot\sigma\cdot K_\mathrm{e}^{i-1}[\mathrm{M}]^i\right)-\sigma\cdot[\mathrm{M}]+[\mathrm{M}]
\end{aligned} \tag{S3}$$

With standard expressions for converging series, the summation in Eq. S3 can be solved and the mass-balance equation for the system can be obtained:

$$[\mathrm{M}]_\mathrm{tot} = (1-\sigma)\cdot[\mathrm{M}] + \frac{\sigma\cdot[\mathrm{M}]}{(1-K_\mathrm{e}\cdot[\mathrm{M}])^2} \tag{S4}$$

This equation is solved in Matlab® using a custom written binary search algorithm to obtain the free monomer concentration. The free monomer concentration is then used to calculate the concentration of nucleated aggregates.

The binding constant K_e is rendered temperature-dependent through the van ‘t Hoff expression:

$$K_e = \exp\left(\frac{-\Delta G_e}{R \cdot T}\right) = \exp\left(\frac{-\Delta H_e}{R \cdot T} + \frac{\Delta S}{R}\right) \quad \text{(S5)}$$

Where R is the gas constant, T is the temperature, ΔH_e and ΔS are the enthalpy and entropy of elongation, respectively.

The nucleation penalty NP is related to the cooperativity parameter σ via:

$$\sigma = e^{\frac{-NP}{R \cdot T}} \quad \text{(S6)}$$

The above-described model is fitted to the CD signal at 224 nm. To predict the spectroscopic response, the concentration of every aggregate type (M and P) is multiplied by the molar absorbance or molar ellipticity for the specific aggregate types:

$$\theta = \theta_M \cdot [M] + \theta_P \cdot [P]_{tot} \quad \text{(S7)}$$

where θ is the ellipticity in mdeg, θ_i is the molar ellipticity of species i in mdeg·M·cm^{-1}. The molar ellipticity of the monomers θ_M is fixed at 0. The fit parameters were ΔH_e, ΔS, NP and θ_P.

The differences between the simulated data and the experimental data were combined in a cost vector. Minimization of the cost vector was performed using the Matlab® lsqnonlin function with the Levenberg-Marquardt algorithm to obtain optimal values for the thermodynamic parameters of the supramolecular polymerization. To ensure that the solution is at the global minimum, the fits were performed with a minimum of 500 initial parameter sets. The initial parameter sets were defined using a Latin Hypercube Sampling method, implemented with the Matlab® function lhsdesign. To ensure reasonable values of the set of starting parameters in the fitting procedure, ΔG_e was sampled between −40 and −10 kJ/mol, ΔS between −150 and −50 J/mol·K, ΔG_n between −20 and −5 kJ/mol and θ_P between $-1.2 \cdot 10^6$ and $-0.8 \cdot 10^6$ mdeg·M·cm^{-1}. The final fitting parameters that resulted in the lowest norm of the residual cost vector were selected as the best fit.

Table S1. Optimized thermodynamic parameters for the supramolecular polymerization of ***S*-BTA-Pnc** determined by fitting the theoretical model for cooperative polymerization to the experimental CD cooling curves.

	ΔH_e (kJ mol^{-1})	ΔS (kJ mol^{-1} K^{-1})	NP[a] (kJ mol^{-1})	σ[b]
***S*-BTA-Pnc**	-80.5	-0.152	16.85	$9.9 \cdot 10^{-4}$

[a] *NP* indicates the enthalpic nucleation penalty. [b] The cooperativity parameter (σ) is calculated at 20 °C.

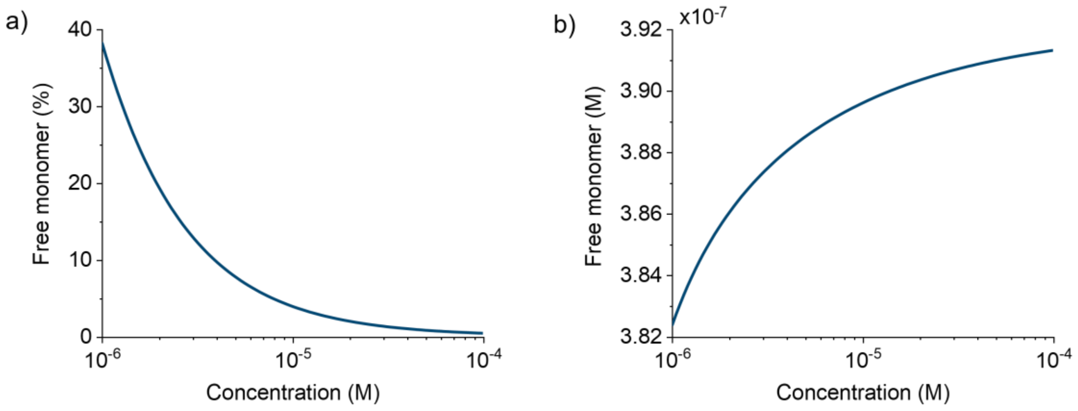

Figure S14. Estimation of a) molar ratio and b) absolute concentration of free monomer in ***S*-BTA-Pnc** solutions in MCH at 20 °C as a function of the total concentration. Calculated with the optimized fitting parameters shown in Table S1.

3.3 Atomic force microscopy

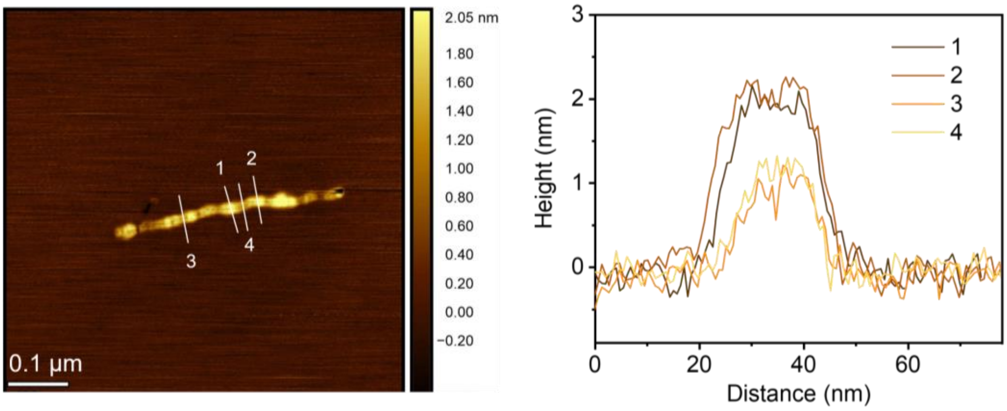

Figure S15. Left) AFM height images of a single fiber from a spin-coated sample (spinning rate: 1000 rpm) prepared by dynamic loading of 10 µM ***S*-BTA-Pnc** solution in MCH on freshly cleaved mica. Right) Height profile of the ***S*-BTA-Pnc** assembly along the lines indicated in the AFM image.

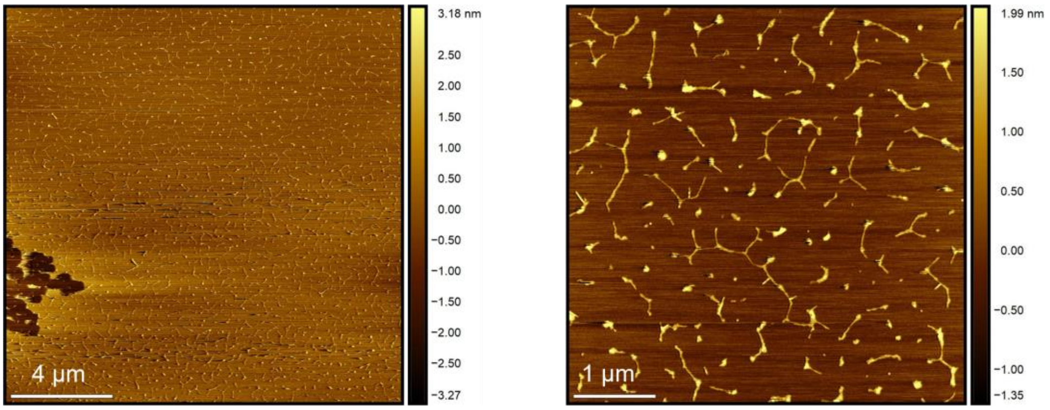

Figure S16. AFM height images of a spin-coated sample (spinning rate: 1000 rpm) prepared by static loading of 10 µM ***S*-BTA-Pnc** solution in MCH on freshly cleaved mica. Branched structures are formed as a result of drying effects. The formation of shorter assemblies than the ones typically afforded by supramolecular polymerization of ***S*-BTA** is in line with the lower cooperativity found for ***S*-BTA-Pnc**, being likely related to the steric hindrance of bulky TIPS-Pnc pendants.

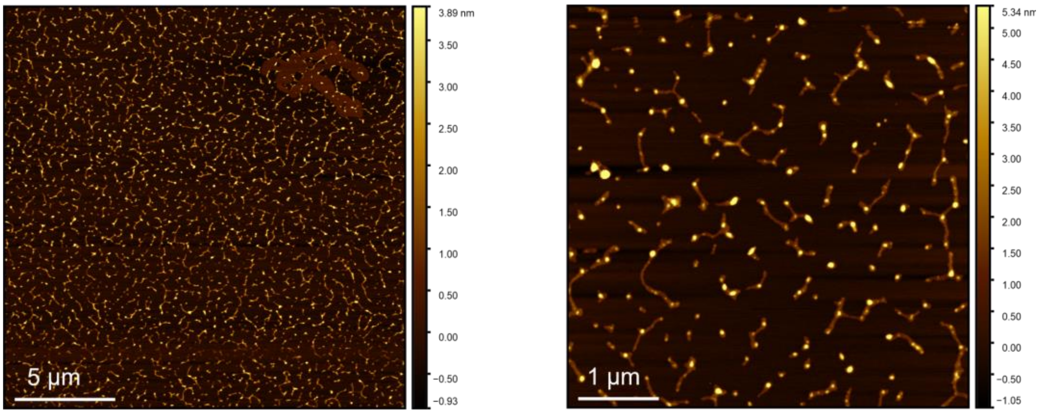

Figure S17. AFM height images of a spin-coated sample (spinning rate: 1000 rpm) prepared by static loading of 10 μM **a-BTA-Pnc** solution in MCH on freshly cleaved mica. Branched structures are formed as a result of drying effects.

4. Photophysical characterization

4.1 Steady-state emission spectroscopy

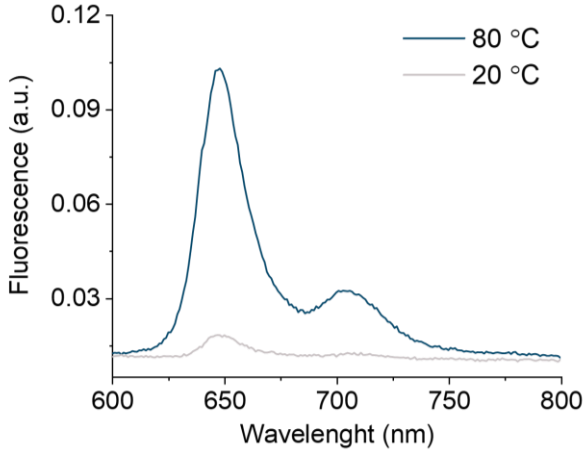

Figure S18. Steady-state emission spectra (λ_{ex} = 321 nm) of 10 μM **a-BTA-Pnc** in MCH at 80 °C (monomerically dissolved state) and 20 °C (assembled state).

4.2 Transient absorption spectroscopy

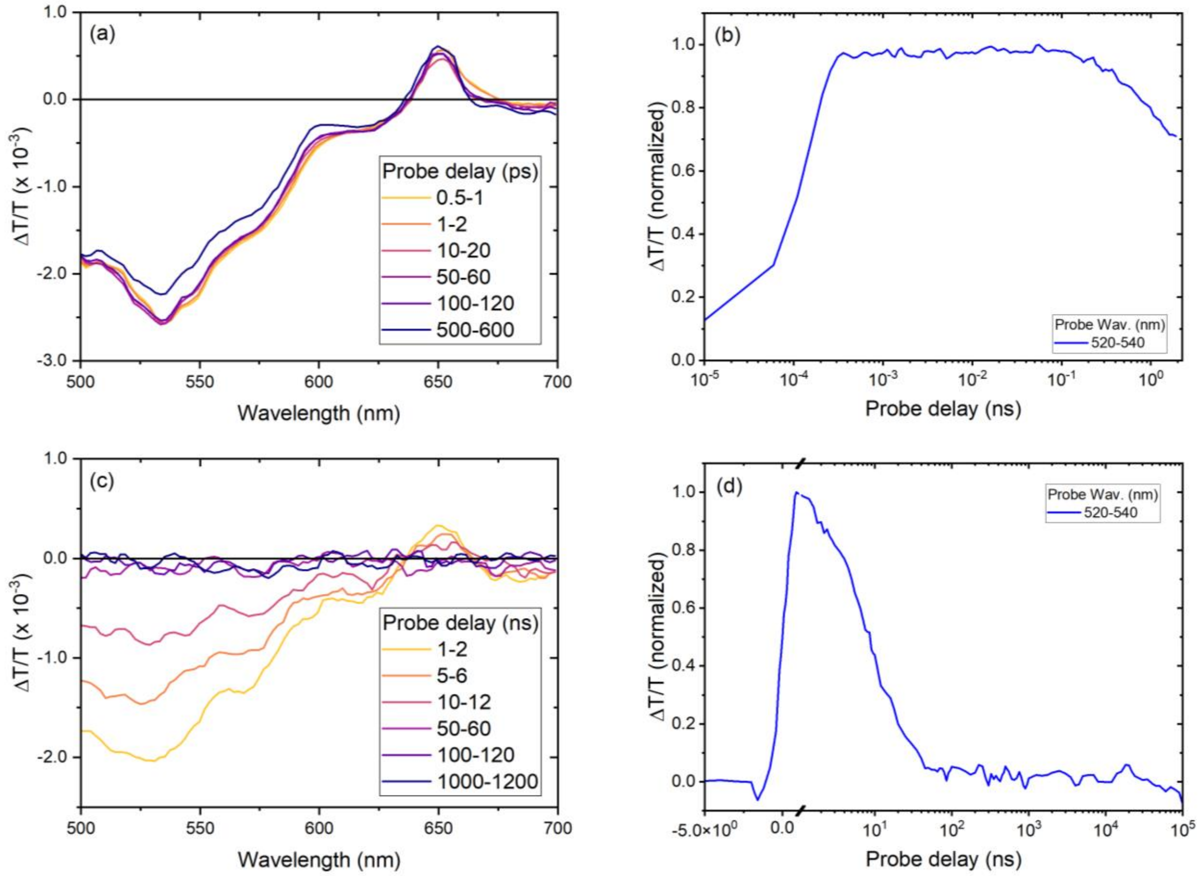

Figure S19. a,c) TA spectra in the (a) ps and (c) ns timescales of 10 μM ***S*-BTA-Pnc** in $CHCl_3$ at room temperature (λ_{ex} = 660 nm.). b,d) Kinetics monitored at 520-540 nm in the

(b) ps and (d) ns timescales of 10 uM ***S*-BTA-Pnc** in $CHCl_3$ at room temperature. In contrast to measurements on ***S*-BTA-Pnc** supramolecular polymers in MCH, no evidence of SF is observed for molecularly dissolved ***S*-BTA-Pnc** in $CHCl_3$. Similar conclusions can be drawn for molecularly dissolved **a-BTA-Pnc** monomers in $CHCl_3$.

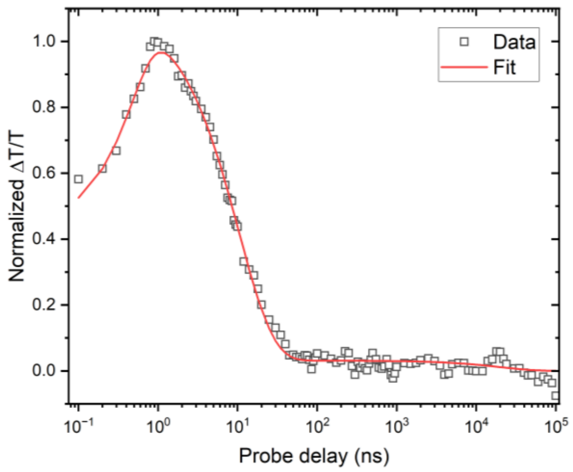

Figure S20. Kinetics monitored at 520-540 nm in the ns timescales of 10 uM ***S*-BTA-Pnc** in $CHCl_3$ at room temperature. The data is best fit with a biexponential decay with a predominant component (>99%) of about 10.5 + 0.5 ns and a second minor component > 10 us. The first short component is similar to the PL lifetime measured for the same solution by TCSPC while the minor longer component is reminiscent of triplets generated through inter-system crossing in TIPS-Pnc in $CHCl_3$.

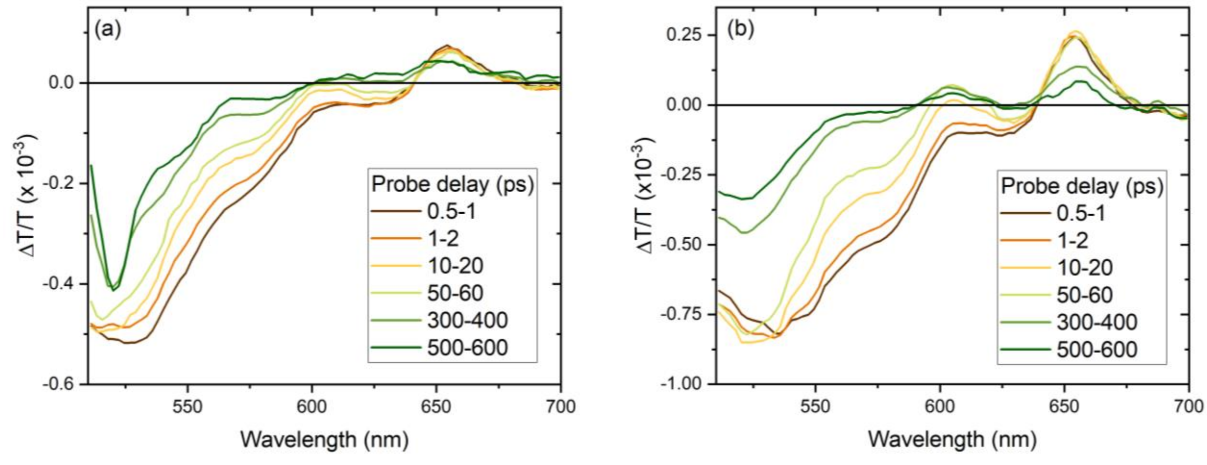

Figure S21. TA spectra in the ps timescale of 10 μM a) ***S*-BTA-Pnc** and b) **a-BTA-Pnc** in MCH at room temperature (assembled state, λ_{ex} = 660 nm). Features attributed to triplet excited states can be seen to evolve in tens of ps – a PIA around 517 nm and recovery of ground state bleach around 600 nm.

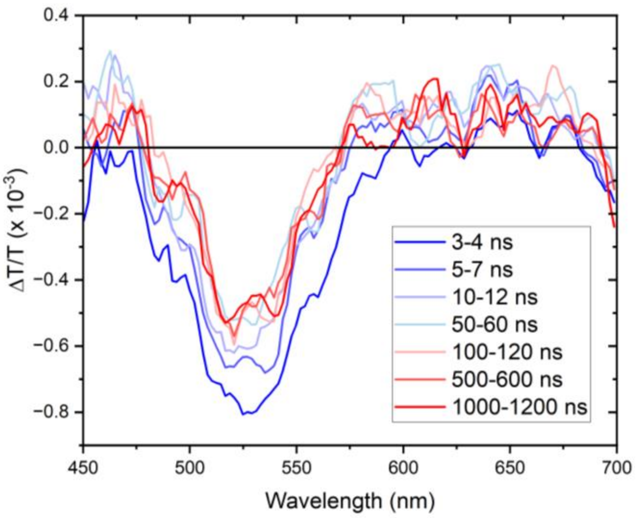

Figure S22. TA spectra in the ns timescale of 10 μM **a-BTA-Pnc** in MCH at room temperature (assembled state, λ_{ex} = 355 nm).

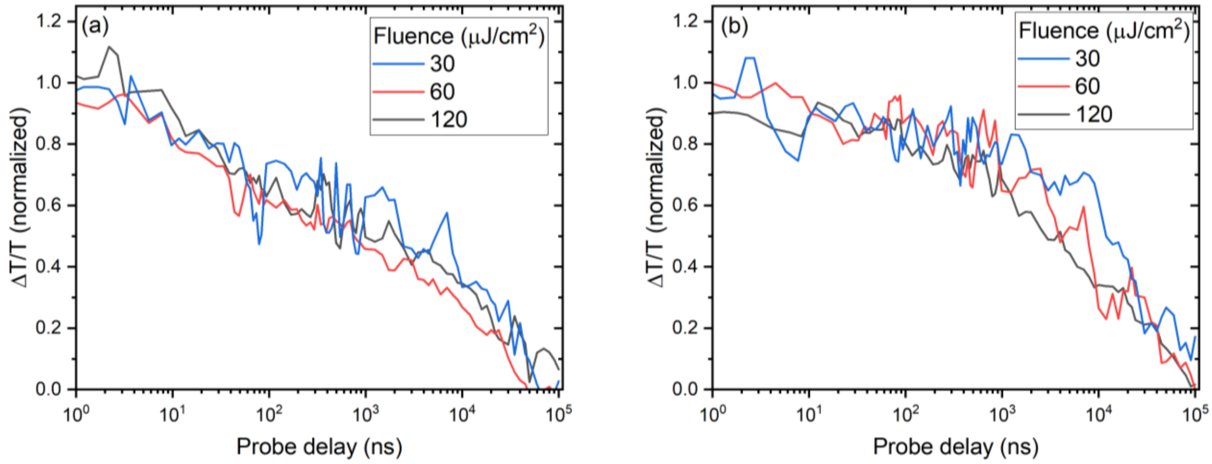

Figure S23. Effect of excitation fluence on the triplet dynamics of (a) ***S*-BTA-Pnc** and (b) **a-BTA-Pnc** supramolecular polymers in MCH at room temperature.

4.3 Triplet yield quantification

To quantify the triplet yield from the transient absorption spectroscopy data, we follow the method reported by Walker *et al.*[5] Briefly, the number of photogenerated singlet states per unit area can be obtained if the absorbed photon flux and the pump beam diameter are known (Eq. S8). Then, the number of triplets generated in the system can be calculated from the magnitude of the TA signal and the extinction coefficient of the triplet excited states of TIPS-Pnc monomer (Eq. S9).

$$n_S = \frac{photons\ absorbed}{area} = \frac{power\ absorbed}{rep.rate} * \frac{\lambda}{hc} * \frac{1}{Area} \tag{S8}$$

$$n_T = \frac{\log((\Delta T/T)_T + 1)}{\varepsilon_T} \tag{S9}$$

Here, $rep.rate$ is the frequency of on-off pump pulses (500 Hz), h is Planck's constant, c is the speed of light, $area$ is the area of the excitation spot, $(\Delta T/T)_T$ is the measured TA response of the triplets and ε_T is the extinction coefficient of the triplet excited state at the relevant wavelength. The triplet yield of the system is then defined as $100 * (n_T/n_S)$. $(\Delta T/T)_T$ can be conveniently monitored in the wavelength region where the contribution from the photoexcited singlet state is minimal (635-640 nm for ***S*-BTA-Pnc** and **a-BTA-Pnc**). The values obtained for the maximum triplet yield of ***S*-BTA-Pnc** and **a-BTA-Pnc** solutions in MCH are reported in Table S2.

Table S2. Parameters for calculating triplet quantum yield (TQY) and calculated TQY values for 10 μM ***S*-BTA-Pnc** and **a-BTA-Pnc** in MCH.

	Power absorbed (μW)	Rep. rate (Hz)	λ (nm)	Area (cm²)	$(\Delta T/T)_T$	ε_T ($M^{-1}\,cm^{-1}$)	TQY (%)
***S*-BTA-Pnc**	29.2	9500	660	$4.25 \cdot 10^{-3}$	$3.5 \cdot 10^{-5}$	3000-4000	114 ± 16
***a*-BTA-Pnc**	35	9500	660	$4.25 \cdot 10^{-3}$	$6.0 \cdot 10^{-5}$	3000-4000	162 ± 22

As shown in Table S2, the triplet yield for ***S*-BTA-Pnc** and **a-BTA-Pnc** fibers in MCH easily exceeds 100%, confirming that SF is the dominant non-radiative pathway for Pnc moieties in the fibers. The concentration of photoexcited singlet states is about 0.3-0.4% of the total monomer concentration (Equation S8), which suggests that for a fiber containing 1000 monomers, 3-4 singlet excited states are generated.

4.4 Global fits

The transient absorption spectra of ***S*-BTA-Pnc** and ***a*-BTA-Pnc** supramolecular polymers in MCH show a time-dependent evolution consistent with the formation of triplets through SF. As the singlet and triplet spectra overlap significantly, the singlet and triplet signatures need to be deconvolved to monitor the evolution of individual species populations over time. We employ global fitting of the TA data sets to identify the distinct spectral shapes involved and extract the respective relative populations of species at any time. The global fits to the data are performed employing principal component analysis through a custom-built graphical user interface on Matlab. The goodness of the fit was evaluated by monitoring the sum of squared errors of prediction (SSE) calculated from the residuals.

Global fits of the TA data in the ps-timescales reveals that only two distinct spectra (two distinct species) are necessary to explain the data (Figure S24). The system of equations that best fit to the data at ps-timescales are:

$$\frac{d[species1]}{dt} = -(k_1 + k_2)\,[species\ 1] \tag{S10}$$

$$\frac{d[species\ 2]}{dt} = k_2[species\ 1] - k_3[species\ 2] \tag{S11}$$

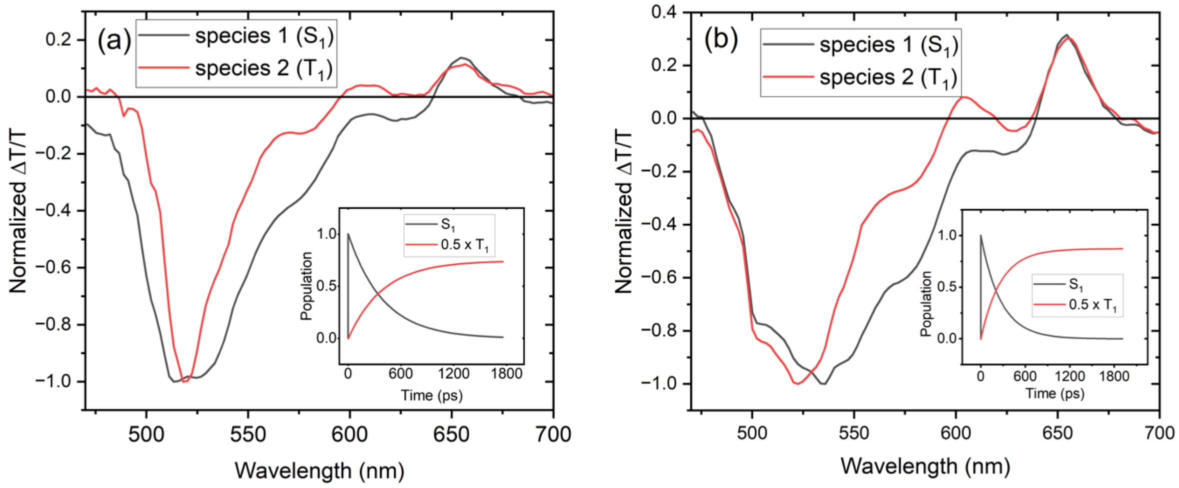

Figure S24. Deconvolved singlet and triplet spectra obtained after global fitting the TA data at ps-timescales of (a) ***S*-BTA-Pnc** and (b) ***a*-BTA-Pnc** supramolecular polymers in MCH. Insets show the extracted evolution of the singlet and triplet species at sub-ns timescales.

Species 1 is generated at photoexcitation and has the characteristic signatures of singlet state in pentacene, thus, is assigned to the S_1 state. Species 2 forms from species 1 in a timescale of few 100's of ps and has features of the triplet state in pentacene (PIA around 520 nm and recovery of GSB around 600 nm). Species 2 is thus assigned as pair of triplets $(T_1 + T_1)$ that form because of SF in the polymers. It should be noted that the as the spectra of the (TT) intermediate and the free triplet $(T_1 + T_1)$ are indistinguishable in TA, TA data

alone cannot be used to adequately determine the difference in the population of the intermediates and free triplets.

As shown in Figure S21, S_1 to $(T_1 + T_1)$ conversion in ***S*-BTA-Pnc** and ***a*-BTA-Pnc** supramolecular polymers in MCH is complete within a few hundreds of ps. The values of SF rate (k_2) obtained from global fitting for ***S*-BTA-Pnc** and ***a*-BTA-Pnc** polymers are 1.88 ns^{-1} and 3.28 ns^{-1}, respectively. The other rates, k_1 and k_3 are smaller than 0.5 ns^{-1} (slower rates cannot be resolved due to the limited time window of the ps-TA measurements). Two conclusions can be made from the global fit of the TA data in ps-timescales: (1) SF is efficient in both ***S*-BTA-Pnc** and ***a*-BTA-Pnc** polymers (k_2>k_1) and (2) SF is slightly faster in ***a*-BTA-Pnc** (k_2 = 3.28 ns^{-1}) polymers compared to ***S*-BTA-Pnc** (k_2 = 1.88 ns^{-1}) polymers. This is consistent the tighter packing of BTA cores within the supramolecular backbone evidenced by chiroptical studies (*vide supra*).

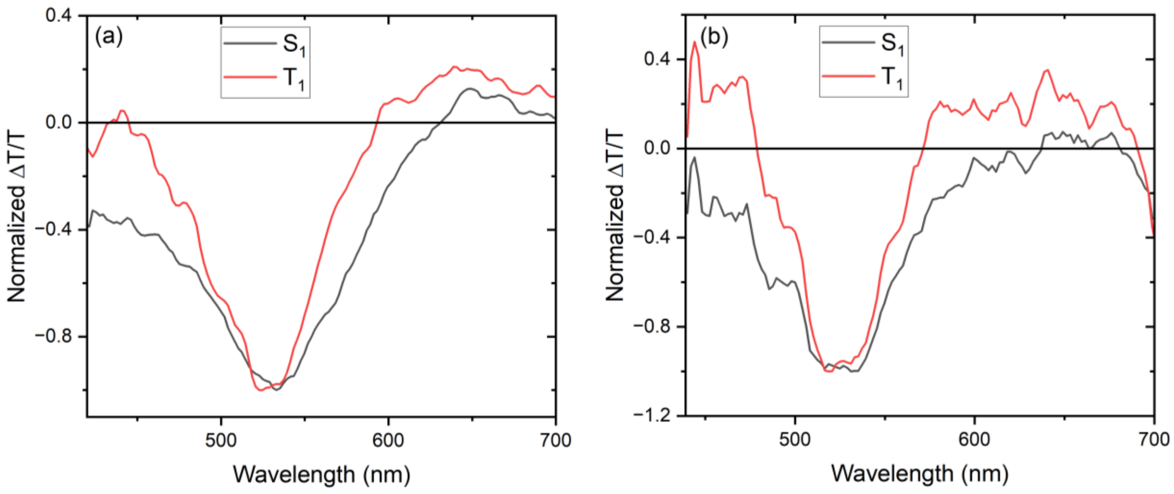

Figure S25. a,b) Deconvolved singlet and triplet spectra obtained after global fitting the TA data at ns-timescales of (a) ***S*-BTA-Pnc** and (b) ***a*-BTA-Pnc** supramolecular polymers in MCH.

In the ns-timescale, global fitting the TA data can be used to deconvolve the residual singlet signal present within the first-few ns (instrument response function of 2-5 ns) from the triplet features. For timescales longer than 5 ns, only triplet species are present in the ns-TA data that have lifetimes of about 15-20 μs. To analyze the evolution of triplet decay kinetics in the fibers across ns-timescales, we develop a Monte-Carlo algorithm of triplet dynamics in the supramolecular fibers.

4.5 Simulation of triplet dynamics in fibers

To capture the effect of the dynamic nature of the inter-pentacene interaction within the fibers on the observed triplet dynamics, we developed a full Monte Carlo simulation of triplet formation, hopping and annihilation.

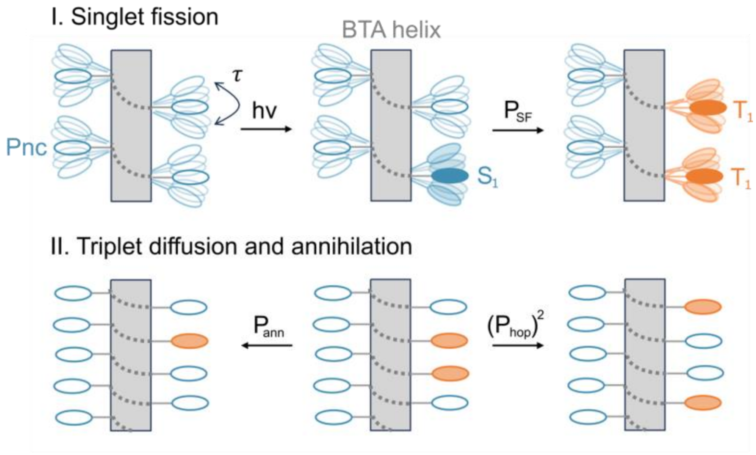

Figure S26. Schematic of the simulation model for triplet formation, hopping and annihilation within the supramolecular polymers.

The fibers are modelled as one-dimensional rods with pendant Pnc moieties attached at regularly spaced intervals (Figure S26). In the simulations, we consider 1000 monomer units per fiber. To sample a statistical number of fibers, we consider 1000 fibers in the simulation and run the simulation dynamics for a total of 100000 time-steps. As discussed in Section 4.3, for the excitation fluences reported here, 2-4 excitations are generated in a fiber incorporating 1000 monomers. Thus, 3 photoexcited singlet excited states per fiber (each separated by 330 monomers) are considered at the start of the simulation.

The dynamic nature of the inter-pentacene interaction within the fibers is modelled by using an average frequency τ. τ controls the rate at which two Pnc moieties come close together for a SF event, hopping event and/or annihilation event to happen. The probability that a SF event occurs when two Pnc moieties interact with one Pnc in the singlet excited state and the other in the ground state is defined as P_{SF} (Eq. S12). The probability that a triplet excited state is transferred to an adjacent molecule in the ground state is defined as P_{hop} (Eq. S13). The probability that the interaction of two adjacent Pnc in their triplet excited states results in the deactivation of one of the triplet excited states to its ground state is defined as P_{ann} (Eq. S14).

$$S_1 + S_0 \xrightarrow{P_{SF}} T_1..T_1 \tag{S12}$$

$$T_1 \cdot\cdot T_1 \xrightarrow{P_{hop}} T_1 + T_1 \tag{S13}$$

$$T_1 \cdot\cdot T_1 \xrightarrow{P_{ann}} T_1 + S_0 \tag{S14}$$

$$S_1 \xrightarrow{k_1} S_0 \tag{S15}$$

$$T_1 \xrightarrow{k_2} S_0 \tag{S16}$$

The rate of unimolecular decay of the singlet excited state and triplet excited state to the ground state is defined as k_1 and k_2, respectively. k_1 is obtained from the time correlated singlet photon counting (TCSPC) experiments on dilute solution of ***S*-BTA-Pnc** in chloroform (k_1 = (10-13 ns)$^{-1}$), while k_2 is approximated from previously reported values in literature (k_2 = (15 μs)$^{-1}$).[6] Varying k_1 and k_2 up to ±20% of their values has no significant effect on the triplet dynamics, the key parameters affecting triplet dynamics being P_{SF}, P_{hop} and P_{ann}.

4.5.1 Separation between generated excitons

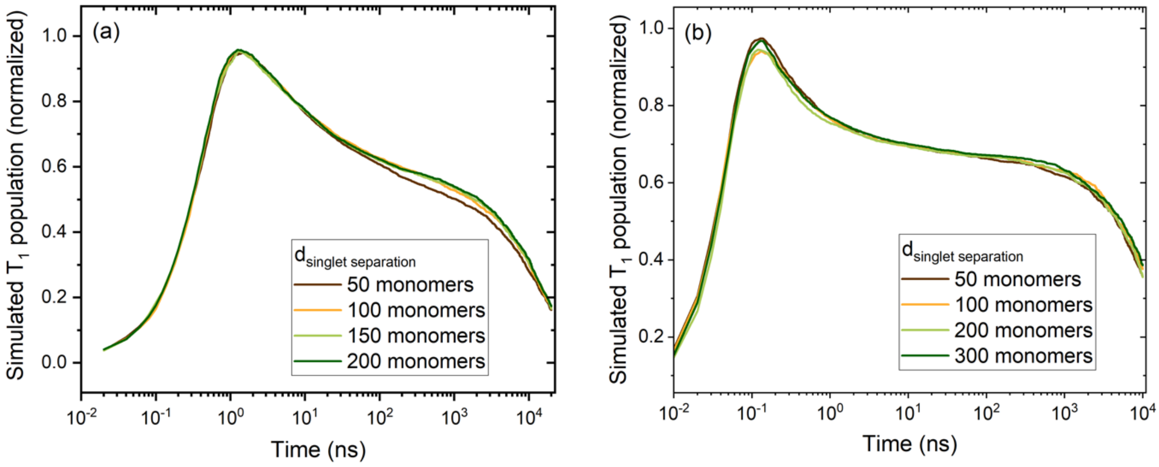

Figure S27. Simulated population of triplets in supramolecular polymers for different separation between photogenerated singlet excitons for (a) τ = 0.05 ps^{-1}, P_{SF} = 0.1, P_{hop} = 0.075, P_{ann} = 0.0075 and (b) τ = 0.1 ps^{-1}, P_{SF} = 0.4, P_{hop} = 0.3, P_{ann} = 0.09. Triplet kinetics are affected by non-geminate annihilation only if pairs of triplet excitons are generated by SF about 50-100 monomers apart.

To investigate whether the annihilation events affecting triplet dynamics are geminate or non-geminate in nature, we simulate the triplet dynamics in supramolecular fibers by varying the distance between photogenerated singlet excited states. As shown in Figure S27a, triplet dynamics are affected by non-geminate annihilation only if triplet pairs are generated at most 50 monomers apart for τ, P_{SF}, P_{hop} and P_{ann} of 0.05 ps^{-1}, 0.1, 0.075 and 0.0075, respectively. These values are used to reproduce triplet dynamics in ***S*-BTA-Pnc** fibers. For τ, P_{SF}, P_{hop} and P_{ann} of 0.1 ps^{-1}, 0.4, 0.3 and 0.09, respectively (used to reproduce triplet dynamics in **a-BTA-Pnc** fibers), non-geminate annihilation affects triplet dynamics for triplet pairs generated at most 100 monomers apart (Figure S27b). For highest excitation fluences reported in this work, triplet pairs are estimated to be generated at least 200-300 monomers apart (see section 4.3) – as such, annihilation events in the fibers are attributed to geminate annihilation between triplet excitons. The predominantly geminate nature of the annihilation is experimentally confirmed by comparing triplet dynamics at different excitation fluences (Figure S23).

4.5.2 Effect of varying parameters (τ, P_{SF}, P_{hop} and P_{ann})

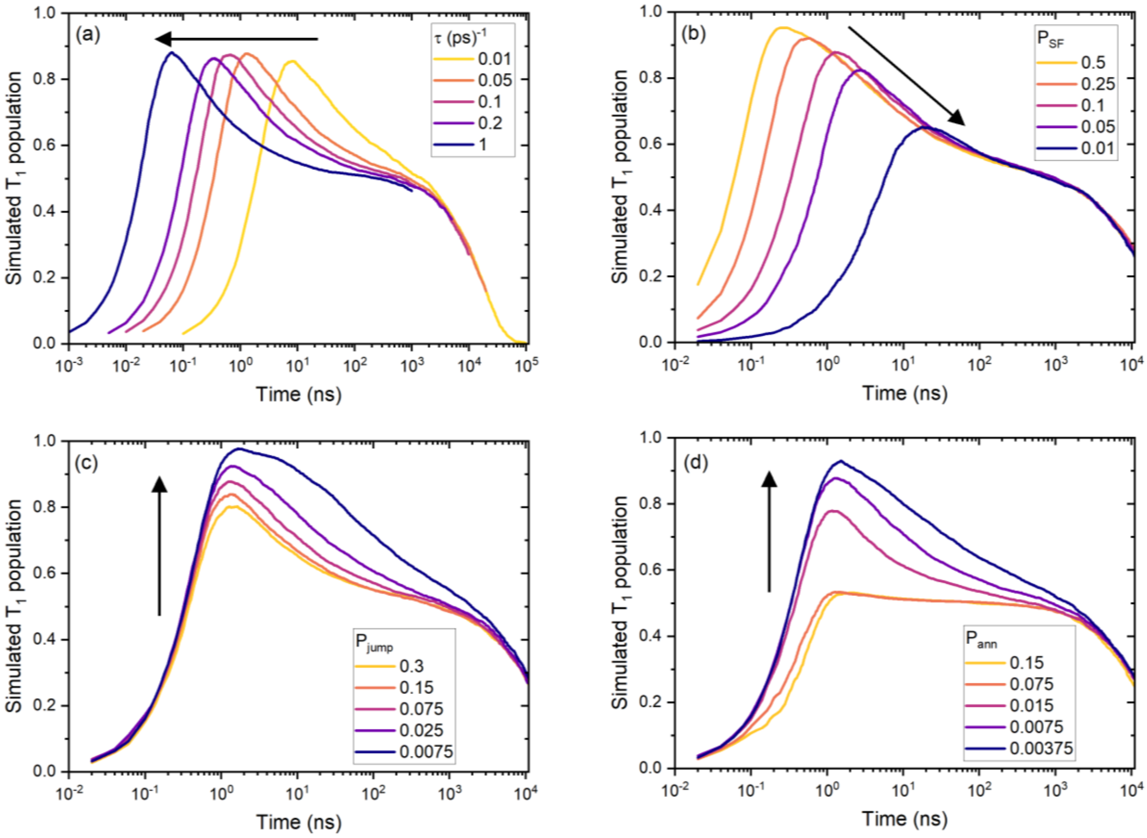

Figure S28. Effect of varying different parameters on the simulated triplet dynamics. Simulated triplet dynamics in fibers for different values of a) frequency (τ) of inter-pentacene interactions with P_{SF} = 0.1, P_{hop} = 0.075 and P_{ann} = 0.0075; b) P_{SF} with τ = 0.05 ps^{-1}, P_{hop} = 0.075 and P_{ann} = 0.0075; c) P_{hop} with τ = 0.05 ps^{-1}, P_{SF} = 0.1 and P_{ann} = 0.0075; d) P_{ann} with τ = 0.05 ps^{-1}, P_{SF} = 0.1 and P_{hop} = 0.075. All spectra are normalized to the maximum possible triplet yield of 200%.

Figure S28 shows the effect of varying different parameters on the simulated triplet dynamics. Increase in the frequency of inter-pentacene interaction (τ) primarily shifts the triplet formation rate such that a higher value of τ implies faster formation of triplets (Figure S28a). This is expected as the faster the inter-pentacene collisions/interactions take place the more likely it is for triplet to form. Expectedly, the triplet formation rate and the overall yield is directly related to the magnitude of P_{SF} (Figure S28b). A decrease in P_{hop} leads to higher triplet yield for a longer period as annihilation events are decreased because triplets do not frequently hop back close to each other when P_{hop} is small (Figure S28c). Similarly, a decrease in P_{ann} results in lower number of annihilation events taking place each time two triplets interact and as such, this leads to a higher percentage of triplet population in the fibers for a longer period (Figure S28d).

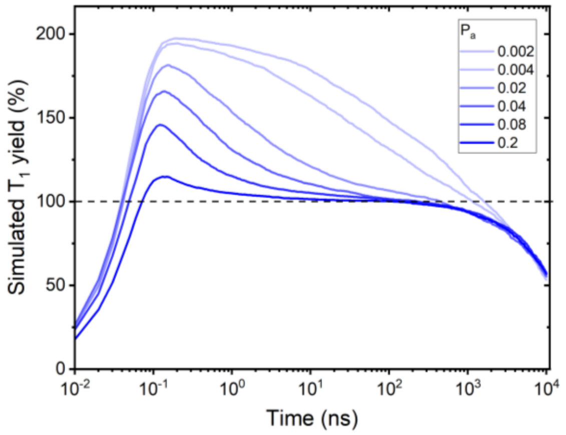

Figure S29. Simulated triplet yield as a function of time for different values of P_{ann}, with τ, P_{SF} and P_{hop} values of 0.1 ps^{-1}, 0.4 and 0.3, respectively. The values of τ, P_{SF} and P_{hop} are set to those used to reproduce the observed kinetic in **a-BTA-PnC** fibers. Triplet yields in the excess of 100% are maintained at least till a 100 ns even for a range of P_{ann}.

5. Molecular modeling

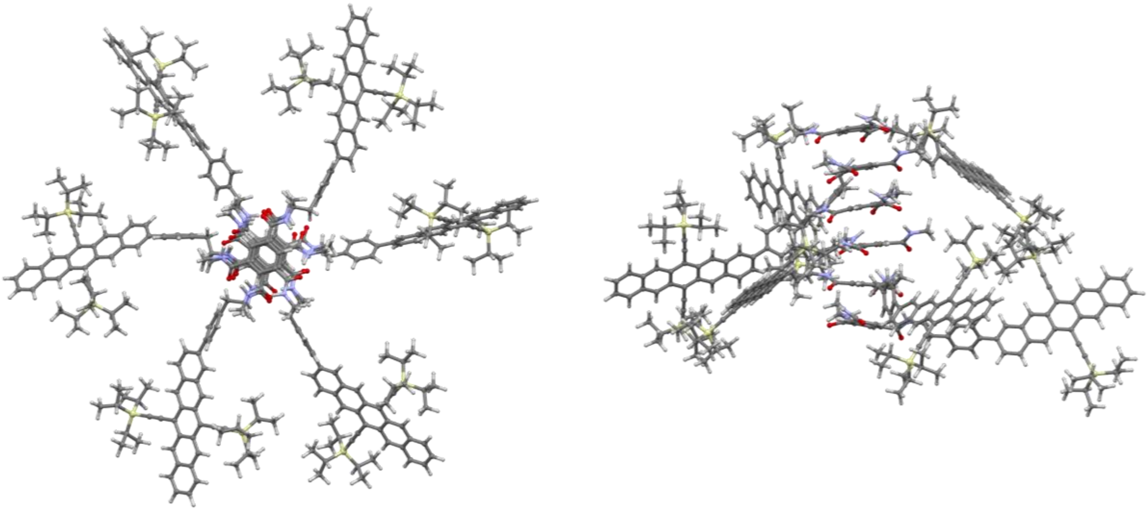

Figure S30. Minimized structure of a BTA-Pnc supramolecular hexamer with methyl side chains obtained from molecular modeling (left: top view; right: lateral view). Conditions: vacuum at room temperature. In Figure 1b (main text), only three monomeric units of the supramolecular hexamer are shown for visualization purposes.

xyz coordinates

O	4.66150	7.36120	-5.15310
C	4.39020	7.88970	-4.07620
N	3.80930	9.09310	-3.98950
C	3.50190	9.95330	-5.12670
C	4.65540	10.90990	-5.46810
C	4.31270	11.87970	-6.58890
C	4.78910	11.65010	-7.87970
C	4.48450	12.54560	-8.90140
C	3.69500	13.67230	-8.64820
C	3.38020	14.63110	-9.73950
C	4.40190	15.15810	-10.53250
C	4.13150	16.06550	-11.55380
C	2.79970	16.46560	-11.81520
C	2.47630	17.37870	-12.83600
C	1.14190	17.79710	-13.13810
C	0.82960	18.73030	-14.18190
C	1.92630	19.31710	-14.96880
C	2.84270	19.81750	-15.60250
Si	4.22480	20.58790	-16.53030
C	3.48000	21.57620	-17.98780
C	2.76930	20.68670	-19.02560
C	2.53600	22.70430	-17.52730
C	5.12750	21.78900	-15.34290
C	5.88120	21.08660	-14.19910
C	6.04730	22.80410	-16.04960
C	5.35990	19.19700	-17.20160
C	6.64570	19.71850	-17.86720
C	5.69200	18.09330	-16.17690
C	-0.52190	19.11100	-14.47520

C	-0.86880	20.02720	-15.51850
C	-2.19010	20.40620	-15.82260
C	-2.49610	21.31370	-16.86750
C	-3.82030	21.65720	-17.13220
C	-4.85520	21.11560	-16.37600
C	-4.58270	20.22150	-15.34260
C	-3.24490	19.85410	-15.05190
C	-2.92030	18.95410	-14.01930
C	-1.58380	18.55620	-13.69770
C	-1.27020	17.63680	-12.64150
C	-2.36550	17.07720	-11.83230
C	-3.29110	16.63230	-11.17090
Si	-4.70720	15.98520	-10.19770
C	-4.78480	16.86520	-8.49570
C	-6.16860	16.78150	-7.82210
C	-4.29890	18.32870	-8.52170
C	-6.31130	16.27990	-11.19850
C	-6.59480	17.76790	-11.48220
C	-6.34970	15.47940	-12.51450
C	-4.42810	14.10890	-9.95870
C	-3.11350	13.77530	-9.22680
C	-5.61050	13.38310	-9.29050
C	0.08080	17.24880	-12.35450
C	0.42920	16.33070	-11.31300
C	1.74980	15.93240	-11.02720
C	2.06000	15.02020	-9.98720
C	3.21310	13.88790	-7.35270
C	3.52320	12.99980	-6.32550
C	4.65890	7.18750	-2.77100
C	5.04350	7.89700	-1.62360
C	5.26010	7.24280	-0.40350
C	5.59260	8.05280	0.82750
N	5.19650	7.53990	2.00060
C	5.38130	8.17710	3.29900
O	6.13880	9.15220	0.73860
C	5.12570	5.84530	-0.35650
C	4.72520	5.11570	-1.48400
C	4.50020	3.62830	-1.37190
N	3.53760	3.11990	-2.15260
C	3.15040	1.71650	-2.19220
O	5.13250	2.94560	-0.56760
C	4.51600	5.79800	-2.69180
H	3.43240	9.36840	-3.08540
H	2.60480	10.53360	-4.90200
H	3.20730	9.38860	-6.01580
H	4.94430	11.47900	-4.58340
H	5.54210	10.33160	-5.73440
H	5.39590	10.78130	-8.09250
H	4.85720	12.35830	-9.89780
H	5.42530	14.86810	-10.34230
H	4.93650	16.46840	-12.15120
H	3.29840	17.77130	-13.41520
H	4.31790	22.04990	-18.50060
H	1.92820	20.15050	-18.58350
H	3.44770	19.94840	-19.45310
H	2.38080	21.27790	-19.85600
H	2.18670	23.30000	-18.37180
H	1.65560	22.30670	-17.02050
H	3.03270	23.38720	-16.83770

H	4.33520	22.37340	-14.87270
H	5.24640	20.36110	-13.68840
H	6.76560	20.56430	-14.56390
H	6.21730	21.80430	-13.44950
H	6.89360	22.31920	-16.53480
H	6.45350	23.52590	-15.33960
H	5.51150	23.37430	-16.80830
H	4.78280	18.70470	-17.98470
H	7.17260	18.91740	-18.38760
H	6.42940	20.49390	-18.60290
H	7.33620	20.13330	-17.13260
H	4.78790	17.65230	-15.75540
H	6.29380	18.46840	-15.35040
H	6.25300	17.28120	-16.64140
H	-0.08650	20.46170	-16.12240
H	-1.69500	21.73590	-17.45690
H	-4.04690	22.34920	-17.93080
H	-5.87730	21.39130	-16.59300
H	-5.38840	19.80230	-14.75720
H	-3.74350	18.54960	-13.45070
H	-4.08700	16.32920	-7.85170
H	-6.14010	17.19070	-6.81120
H	-6.92140	17.34200	-8.37680
H	-6.51820	15.75340	-7.73930
H	-3.27330	18.40240	-8.88560
H	-4.92110	18.95330	-9.16230
H	-4.31150	18.76940	-7.52390
H	-7.13200	15.91290	-10.58110
H	-6.68370	18.34210	-10.56080
H	-5.80240	18.21950	-12.08010
H	-7.53080	17.89740	-12.02730
H	-7.29570	15.62020	-13.03910
H	-5.55090	15.78650	-13.19160
H	-6.24060	14.40940	-12.33610
H	-4.33540	13.69460	-10.96370
H	-2.93920	12.69870	-9.19700
H	-2.25360	14.22340	-9.72620
H	-3.12320	14.13230	-8.19650
H	-5.47060	12.30130	-9.31560
H	-6.55190	13.59590	-9.79770
H	-5.71820	13.66800	-8.24400
H	-0.35160	15.90360	-10.70200
H	1.25780	14.61240	-9.38950
H	2.61020	14.75800	-7.13750
H	3.15330	13.18420	-5.32700
H	5.15400	8.97350	-1.66840
H	4.63140	6.69700	1.98160
H	5.89560	7.49560	3.97610
H	5.96510	9.09760	3.24300
H	4.41120	8.41950	3.73350
H	5.29390	5.31340	0.57200
H	2.93580	3.76940	-2.64230
H	3.00290	1.40440	-3.22590
H	3.89980	1.06160	-1.74460
H	2.21040	1.57760	-1.65830
H	4.22460	5.25250	-3.58090
O	2.31910	9.99830	-1.74330
C	1.99000	9.44940	-0.69180
N	1.91040	10.10020	0.47360

C	2.44650	11.42870	0.74630
C	3.35920	11.40050	1.98170
C	4.13180	12.69260	2.18580
C	5.42160	12.81350	1.66880
C	6.13630	13.99290	1.85800
C	5.57390	15.06270	2.56410
C	6.34710	16.31740	2.77620
C	7.68160	16.26550	3.18770
C	8.42360	17.42590	3.39410
C	7.83210	18.69420	3.18780
C	8.54140	19.89450	3.38090
C	7.97900	21.19410	3.17800
C	8.71730	22.40810	3.37480
C	10.12070	22.33150	3.81260
C	11.28830	22.28740	4.16820
Si	13.04850	22.25310	4.68330
C	13.69650	24.05240	4.68080
C	13.71170	24.69190	3.27910
C	12.93910	24.96400	5.66640
C	13.12970	21.55290	6.46370
C	12.74650	20.06580	6.56840
C	14.46630	21.81610	7.18500
C	14.03330	21.20670	3.41480
C	15.50110	20.97330	3.81380
C	13.36080	19.87370	3.02840
C	8.12570	23.69630	3.15450
C	8.83600	24.92590	3.33400
C	8.27060	26.19730	3.12160
C	9.00640	27.39400	3.30960
C	8.40340	28.63030	3.08610
C	7.07560	28.70440	2.67610
C	6.32920	27.54360	2.48310
C	6.91720	26.27280	2.70400
C	6.20400	25.07260	2.52250
C	6.76260	23.77210	2.73470
C	6.02160	22.55780	2.54580
C	4.61330	22.63800	2.12490
C	3.44580	22.73220	1.77850
Si	1.69860	22.92060	1.24770
C	0.52080	22.45460	2.68720
C	-0.89050	23.05750	2.54450
C	1.08690	22.76230	4.08820
C	1.43200	24.73550	0.70420
C	1.69870	25.75680	1.82720
C	2.24690	25.10970	-0.54870
C	1.41930	21.74750	-0.23580
C	1.70930	20.26950	0.09120
C	0.03080	21.88830	-0.88610
C	6.61590	21.26930	2.75770
C	5.90950	20.03870	2.56790
C	6.48030	18.76710	2.77060
C	5.75260	17.56700	2.56970
C	4.27660	14.93050	3.07360
C	3.55740	13.75220	2.88750
C	1.70410	7.97200	-0.64120
C	2.04600	7.21470	0.48800
C	1.86500	5.82830	0.52130
C	2.27400	5.05470	1.74900
N	1.57410	3.94460	2.01370

C	1.81360	3.05730	3.14500
O	3.17990	5.46500	2.47750
C	1.33090	5.19520	-0.60980
C	0.96570	5.93030	-1.74490
C	0.40490	5.21630	-2.94260
N	-0.58090	5.84930	-3.58990
C	-1.25420	5.34930	-4.78120
O	0.83110	4.10840	-3.26890
C	1.16250	7.31860	-1.75330
H	1.36220	9.66130	1.21020
H	1.66370	12.18220	0.85030
H	3.03530	11.79950	-0.09850
H	2.77990	11.16630	2.87590
H	4.07570	10.58220	1.88940
H	5.87170	11.99350	1.12360
H	7.13210	14.07390	1.44670
H	8.14950	15.30670	3.35910
H	9.45400	17.36330	3.71340
H	9.56970	19.80610	3.69790
H	14.73290	24.00880	5.01760
H	12.70800	24.75060	2.85520
H	14.33320	24.12440	2.58620
H	14.11450	25.70520	3.30870
H	13.37610	25.96300	5.70080
H	11.89110	25.07450	5.38410
H	12.96660	24.56740	6.68160
H	12.37050	22.10370	7.02110
H	11.80320	19.85890	6.06090
H	13.51220	19.42350	6.13340
H	12.62540	19.76290	7.60930
H	15.29410	21.28420	6.71790
H	14.42140	21.49080	8.22540
H	14.71710	22.87670	7.19570
H	14.05100	21.80060	2.50070
H	16.06670	20.53070	2.99280
H	15.99700	21.90720	4.08060
H	15.58190	20.29340	4.66210
H	12.34390	20.02750	2.66540
H	13.31170	19.18070	3.86710
H	13.90950	19.37410	2.22890
H	9.86820	24.89950	3.64910
H	10.03810	27.34000	3.62660
H	8.97010	29.53900	3.23120
H	6.62140	29.67000	2.50600
H	5.29870	27.60480	2.16390
H	5.17730	25.16120	2.20200
H	0.40750	21.37120	2.64010
H	-1.56310	22.67390	3.31290
H	-0.87610	24.14350	2.64030
H	-1.33830	22.81840	1.58090
H	2.03300	22.24700	4.25870
H	1.26260	23.82850	4.23070
H	0.40380	22.43570	4.87330
H	0.37900	24.82700	0.43530
H	1.06080	25.57820	2.69210
H	2.73470	25.71810	2.16600
H	1.50090	26.77600	1.49280
H	2.03880	26.13170	-0.86830
H	3.31980	25.03610	-0.36370

H	2.00990	24.45850	-1.39050
H	2.14940	22.04610	-0.98940
H	1.63300	19.64440	-0.79970
H	2.71780	20.13900	0.48610
H	1.00970	19.87350	0.82790
H	-0.02690	21.31780	-1.81430
H	-0.19380	22.92620	-1.13380
H	-0.75910	21.51860	-0.23230
H	4.87880	20.06480	2.24820
H	4.72590	17.62700	2.24080
H	3.82870	15.74040	3.63030
H	2.56020	13.66290	3.29250
H	2.48090	7.70650	1.34880
H	0.77360	3.73500	1.42540
H	1.97820	2.04120	2.78730
H	2.68330	3.34700	3.73760
H	0.94340	3.05190	3.80190
H	1.19980	4.12020	-0.61690
H	-0.96520	6.68520	-3.16790
H	-1.18140	6.08540	-5.58150
H	-0.83120	4.41200	-5.14650
H	-2.31020	5.18560	-4.56570
H	0.92310	7.89500	-2.63840
O	-6.58370	3.33880	4.15600
C	-7.52440	3.95970	3.66190
N	-8.45420	3.36240	2.90710
C	-8.34880	1.99560	2.40520
C	-7.41610	1.88880	1.18320
C	-7.29010	0.46960	0.65360
C	-6.13140	-0.26990	0.89760
C	-6.01650	-1.56460	0.39700
C	-7.06350	-2.13610	-0.33230
C	-6.94920	-3.51600	-0.86360
C	-5.91870	-3.85500	-1.74220
C	-5.82080	-5.13740	-2.27870
C	-6.76380	-6.13160	-1.92290
C	-6.71560	-7.43850	-2.44440
C	-7.63920	-8.47290	-2.08950
C	-7.58500	-9.79750	-2.63780
C	-6.57680	-10.10640	-3.66470
C	-5.76800	-10.34650	-4.54790
Si	-4.58800	-10.68010	-5.91290
C	-4.64480	-12.55040	-6.30670
C	-4.14060	-13.43290	-5.14890
C	-6.03840	-13.02540	-6.76270
C	-5.17780	-9.69270	-7.44470
C	-5.06540	-8.16560	-7.28630
C	-4.53880	-10.14440	-8.77250
C	-2.82900	-10.19060	-5.33030
C	-1.76490	-10.28110	-6.43830
C	-2.74940	-8.82590	-4.61630
C	-8.49900	-10.82360	-2.22530
C	-8.45770	-12.16050	-2.73410
C	-9.34290	-13.17980	-2.33450
C	-9.27040	-14.49450	-2.85930
C	-10.16880	-15.47040	-2.43250
C	-11.14480	-15.16730	-1.48800
C	-11.23800	-13.88330	-0.95490
C	-10.33780	-12.87090	-1.37190

C	-10.39400	-11.55970	-0.86350
C	-9.50380	-10.51160	-1.25920
C	-9.57920	-9.17820	-0.73570
C	-10.62520	-8.85280	0.24800
C	-11.49370	-8.60080	1.06940
Si	-12.79920	-8.26020	2.31440
C	-14.21610	-7.24470	1.51590
C	-15.54880	-7.34150	2.28390
C	-14.44520	-7.55080	0.02190
C	-13.44190	-9.93370	2.98150
C	-14.05540	-10.83280	1.89070
C	-12.36970	-10.71610	3.76420
C	-11.99240	-7.26230	3.73250
C	-11.37880	-5.92700	3.26820
C	-12.92140	-7.03290	4.93900
C	-8.65870	-8.15550	-1.14080
C	-8.70750	-6.81610	-0.64100
C	-7.79780	-5.80770	-1.01000
C	-7.88190	-4.48970	-0.49820
C	-8.22470	-1.39250	-0.56130
C	-8.33620	-0.09180	-0.07900
C	-7.64590	5.45160	3.83900
C	-8.25340	6.25110	2.86000
C	-8.29650	7.64430	2.98090
C	-8.92710	8.46590	1.88760
N	-9.53980	9.59260	2.26940
C	-10.17640	10.54410	1.36650
O	-8.89630	8.08260	0.71720
C	-7.70390	8.24290	4.10140
C	-7.08920	7.46820	5.09340
C	-6.40870	8.14480	6.25230
N	-6.47080	7.51160	7.42970
C	-5.82770	7.97100	8.65460
O	-5.84350	9.22700	6.09740
C	-7.06890	6.07320	4.95280
H	-9.31870	3.86530	2.72820
H	-9.33780	1.61170	2.14810
H	-8.01600	1.30380	3.18430
H	-7.76920	2.53830	0.38050
H	-6.42570	2.27340	1.43360
H	-5.31920	0.15370	1.47080
H	-5.11770	-2.13400	0.58740
H	-5.19620	-3.10460	-2.03000
H	-5.02660	-5.37930	-2.97020
H	-5.92500	-7.65370	-3.14750
H	-3.96360	-12.71080	-7.14320
H	-4.75700	-13.31310	-4.25670
H	-3.11230	-13.19180	-4.87900
H	-4.15730	-14.48980	-5.41890
H	-6.02470	-14.07660	-7.05380
H	-6.77820	-12.91490	-5.96850
H	-6.39310	-12.45940	-7.62450
H	-6.24290	-9.91230	-7.53130
H	-5.51160	-7.82680	-6.35020
H	-4.02650	-7.83630	-7.30490
H	-5.58170	-7.64690	-8.09510
H	-3.46920	-9.94000	-8.80150
H	-4.98840	-9.62580	-9.62040
H	-4.67790	-11.21160	-8.94500

H	-2.55690	-10.93100	-4.57760
H	-0.75980	-10.17050	-6.02880
H	-1.80070	-11.24310	-6.95050
H	-1.89590	-9.49650	-7.18370
H	-3.44200	-8.77210	-3.77570
H	-2.97700	-7.99830	-5.28650
H	-1.75040	-8.64990	-4.21530
H	-7.70860	-12.42510	-3.46520
H	-8.51330	-14.73400	-3.59210
H	-10.10830	-16.47070	-2.83700
H	-11.83480	-15.93430	-1.16620
H	-11.99670	-13.65160	-0.22120
H	-11.16080	-11.35430	-0.13220
H	-13.89960	-6.20240	1.56330
H	-16.29410	-6.66220	1.86790
H	-15.96700	-8.34740	2.23870
H	-15.43180	-7.08090	3.33490
H	-13.54250	-7.37630	-0.56500
H	-14.74780	-8.58530	-0.13970
H	-15.22350	-6.91350	-0.39990
H	-14.24050	-9.70010	3.68650
H	-14.89030	-10.34340	1.39040
H	-13.32050	-11.09680	1.12920
H	-14.43950	-11.76280	2.31180
H	-12.77480	-11.64020	4.17870
H	-11.52660	-10.98520	3.12580
H	-11.98040	-10.13570	4.60100
H	-11.16380	-7.87590	4.08940
H	-10.84040	-5.43560	4.07980
H	-10.66550	-6.07680	2.45670
H	-12.14250	-5.23300	2.91620
H	-12.37640	-6.59440	5.77610
H	-13.35620	-7.96740	5.29490
H	-13.73720	-6.35250	4.69570
H	-9.47620	-6.54370	0.06610
H	-8.67580	-4.24270	0.19160
H	-9.03560	-1.82170	-1.13230
H	-9.23370	0.47580	-0.27780
H	-8.67570	5.79100	1.97530
H	-9.63010	9.77770	3.26390
H	-9.78710	11.54490	1.55290
H	-10.00770	10.31060	0.31350
H	-11.25190	10.56040	1.54210
H	-7.69460	9.32210	4.19570
H	-7.06680	6.69340	7.50740
H	-5.28980	7.14510	9.11990
H	-5.11360	8.77800	8.48050
H	-6.57870	8.32680	9.35960
H	-6.56450	5.46390	5.69330
O	-11.12990	4.47300	2.76860
C	-11.60020	5.54060	3.16150
N	-12.43380	6.27920	2.41830
C	-12.70920	6.07090	0.99750
C	-12.55080	7.37930	0.20490
C	-12.55420	7.16980	-1.30030
C	-11.35770	7.23790	-2.01470
C	-11.36030	7.03600	-3.39280
C	-12.55410	6.76350	-4.07080
C	-12.55840	6.54240	-5.54300

C	-11.85460	7.39800	-6.39490
C	-11.85630	7.21020	-7.77560
C	-12.57860	6.13600	-8.34700
C	-12.61450	5.90550	-9.73550
C	-13.33350	4.82910	-10.34650
C	-13.36190	4.60720	-11.76360
C	-12.62730	5.52630	-12.64740
C	-12.02880	6.28270	-13.39700
Si	-11.14500	7.40200	-14.55060
C	-11.35700	6.70830	-16.31980
C	-10.70150	5.32750	-16.51490
C	-12.82940	6.65850	-16.77310
C	-11.96840	9.12740	-14.43730
C	-11.75310	9.83540	-13.08740
C	-11.61710	10.07330	-15.60260
C	-9.28730	7.41860	-14.07980
C	-8.45590	8.43320	-14.88450
C	-9.01240	7.57200	-12.57020
C	-14.08890	3.51440	-12.34320
C	-14.13470	3.26210	-13.75140
C	-14.84460	2.19560	-14.33510
C	-14.86820	1.97790	-15.73560
C	-15.58580	0.90800	-16.26700
C	-16.28510	0.04230	-15.43150
C	-16.27840	0.23260	-14.05110
C	-15.55760	1.31330	-13.48380
C	-15.52200	1.54640	-12.09620
C	-14.80770	2.62630	-11.48630
C	-14.78430	2.85150	-10.06960
C	-15.53310	1.94010	-9.18880
C	-16.16730	1.17240	-8.48140
Si	-17.13410	-0.00480	-7.45690
C	-18.68350	0.87250	-6.74610
C	-19.79440	-0.10220	-6.30870
C	-19.26950	1.95960	-7.66980
C	-17.62460	-1.49320	-8.55460
C	-18.50200	-1.10350	-9.76010
C	-16.40810	-2.30850	-9.03380
C	-15.99890	-0.59050	-6.03420
C	-15.48210	0.56170	-5.15050
C	-16.61750	-1.69740	-5.16090
C	-14.05340	3.94130	-9.49010
C	-14.00610	4.19150	-8.08220
C	-13.29450	5.25680	-7.49800
C	-13.27270	5.47530	-6.09820
C	-13.74800	6.70370	-3.34310
C	-13.75140	6.90700	-1.96590
C	-11.18630	6.12650	4.49010
C	-11.20610	7.51230	4.71000
C	-10.64610	8.07640	5.86140
C	-10.54450	9.57620	5.97810
N	-10.53980	10.07790	7.21810
C	-10.39210	11.49470	7.53710
O	-10.47160	10.27730	4.96850
C	-10.08590	7.22570	6.82480
C	-10.05950	5.83760	6.63850
C	-9.31550	4.97180	7.62310
N	-9.74160	3.70970	7.74780
C	-9.12220	2.70340	8.60600

O	-8.36900	5.42860	8.26360
C	-10.62670	5.29950	5.47300
H	-12.93490	7.01370	2.88850
H	-13.68090	5.60730	0.81640
H	-12.01100	5.34410	0.56970
H	-13.33530	8.08740	0.47420
H	-11.61800	7.87000	0.48850
H	-10.42710	7.44840	-1.50470
H	-10.42790	7.08390	-3.93660
H	-11.30600	8.23170	-5.98090
H	-11.30940	7.88530	-8.41810
H	-12.06050	6.59210	-10.35810
H	-10.84020	7.39940	-16.98680
H	-11.14980	4.57570	-15.86350
H	-9.63260	5.35680	-16.30320
H	-10.81100	4.97730	-17.54220
H	-12.91530	6.33010	-17.80980
H	-13.41310	5.96880	-16.16150
H	-13.30270	7.63840	-16.70780
H	-13.04000	8.93670	-14.51430
H	-11.99010	9.17920	-12.24890
H	-10.72290	10.17240	-12.97260
H	-12.39150	10.71540	-12.99870
H	-10.56350	10.35020	-15.60080
H	-12.19270	10.99820	-15.54460
H	-11.83980	9.62230	-16.56950
H	-8.91200	6.43030	-14.34620
H	-7.38790	8.29150	-14.71320
H	-8.62680	8.33030	-15.95670
H	-8.69300	9.45910	-14.60170
H	-9.53280	6.81070	-11.98790
H	-9.32160	8.54600	-12.19410
H	-7.94890	7.46510	-12.35230
H	-13.59990	3.91580	-14.42400
H	-14.32580	2.64770	-16.38740
H	-15.60010	0.74780	-17.33550
H	-16.83680	-0.78360	-15.85770
H	-16.82200	-0.44210	-13.40540
H	-16.07260	0.85760	-11.47390
H	-18.34660	1.39120	-5.84820
H	-20.60820	0.42520	-5.80890
H	-20.22570	-0.62960	-7.16000
H	-19.42490	-0.85190	-5.61040
H	-18.53180	2.72920	-7.90040
H	-19.61860	1.54500	-8.61530
H	-20.11620	2.46390	-7.20220
H	-18.22280	-2.15620	-7.92850
H	-19.42700	-0.62210	-9.44450
H	-17.98170	-0.41680	-10.42900
H	-18.78520	-1.97960	-10.34510
H	-16.71650	-3.18000	-9.61310
H	-15.75070	-1.71040	-9.66700
H	-15.81640	-2.67710	-8.19540
H	-15.12350	-1.02690	-6.51750
H	-14.76280	0.20240	-4.41320
H	-14.97650	1.32350	-5.74530
H	-16.29250	1.04720	-4.60590
H	-15.88110	-2.10870	-4.46910
H	-16.98730	-2.52680	-5.76470

H	-17.44600	-1.32110	-4.56090
H	-14.54100	3.53650	-7.41140
H	-13.81460	4.79590	-5.45720
H	-14.68150	6.50890	-3.85130
H	-14.68460	6.86050	-1.42350
H	-11.57600	8.17840	3.94080
H	-10.70300	9.43920	7.97760
H	-9.67970	11.62170	8.35240
H	-10.03040	12.08140	6.69020
H	-11.34900	11.91220	7.85090
H	-9.59140	7.64340	7.69310
H	-10.57070	3.44830	7.24210
H	-8.87210	1.81820	8.02060
H	-8.20360	3.05580	9.07960
H	-9.81230	2.40660	9.39610
H	-10.55190	4.23740	5.27570
O	0.18280	9.12880	2.51490
C	-0.39410	8.05520	2.69000
N	-0.33440	7.38870	3.84700
C	0.59500	7.65810	4.93630
C	1.14580	6.34540	5.51300
C	2.29900	6.55240	6.48020
C	3.60890	6.31710	6.06120
C	4.66410	6.49950	6.95100
C	4.42330	6.92290	8.26290
C	5.54930	7.11050	9.21640
C	6.52900	6.12490	9.36000
C	7.58200	6.27550	10.25920
C	7.68020	7.44450	11.04990
C	8.72110	7.64010	11.97650
C	8.85520	8.80980	12.78950
C	9.91980	8.99120	13.73340
C	10.91940	7.92390	13.89750
C	11.73840	7.02840	14.03440
Si	12.98060	5.69620	14.24530
C	14.28080	6.30490	15.50760
C	15.08110	7.52560	15.01430
C	13.67730	6.59800	16.89530
C	12.08110	4.15800	14.94760
C	11.08390	3.51420	13.96740
C	13.02370	3.08940	15.53530
C	13.82000	5.37540	12.55260
C	14.79120	4.18190	12.56160
C	12.84230	5.26670	11.36500
C	10.03450	10.18280	14.52350
C	11.08870	10.39840	15.46680
C	11.21760	11.56310	16.24660
C	12.27780	11.74220	17.17020
C	12.36440	12.91360	17.91970
C	11.41380	13.91890	17.77080
C	10.36130	13.76910	16.86990
C	10.24880	12.58780	16.09470
C	9.20420	12.39110	15.17160
C	9.05790	11.21370	14.37110
C	7.98690	11.02790	13.43450
C	6.97970	12.09010	13.28040
C	6.16300	12.99020	13.15840
Si	4.97230	14.37590	12.98100
C	3.27820	13.88310	13.73190

C	2.39160	15.09150	14.09120
C	3.37810	12.92590	14.93710
C	5.70070	15.90630	13.86850
C	5.94040	15.68480	15.37460
C	6.99090	16.42360	13.20370
C	4.78770	14.73470	11.11210
C	4.29670	13.51930	10.30170
C	3.92570	15.97110	10.79720
C	7.87440	9.83750	12.64170
C	6.82030	9.61930	11.69850
C	6.70060	8.45820	10.91040
C	5.64030	8.27300	9.98870
C	3.10570	7.16050	8.66960
C	2.04690	6.97380	7.78530
C	-1.15400	7.38320	1.57420
C	-1.25630	5.98540	1.50820
C	-1.88370	5.34640	0.43380
C	-1.93320	3.84080	0.38910
N	-3.00890	3.30100	-0.19490
C	-3.22040	1.87330	-0.38580
O	-1.03340	3.16340	0.89070
C	-2.41610	6.13270	-0.59710
C	-2.33640	7.53050	-0.55200
C	-2.89000	8.34320	-1.68810
N	-3.49140	9.48960	-1.34910
C	-4.06550	10.43930	-2.29250
O	-2.79440	7.94280	-2.84840
C	-1.70120	8.14560	0.53660
H	-1.03690	6.67060	4.00930
H	0.16080	8.28580	5.71650
H	1.44540	8.24940	4.58220
H	0.34830	5.78690	6.00550
H	1.47950	5.69920	4.69900
H	3.81020	5.98900	5.04980
H	5.67510	6.31480	6.61710
H	6.46510	5.21960	8.77340
H	8.32720	5.49940	10.35990
H	9.45080	6.84930	12.06180
H	14.99460	5.49030	15.63450
H	14.43240	8.38530	14.83920
H	15.60750	7.31110	14.08420
H	15.83510	7.82740	15.74260
H	14.44970	6.87300	17.61490
H	12.96050	7.41970	16.85400
H	13.15860	5.72820	17.29920
H	11.48820	4.53240	15.78350
H	10.41060	4.25650	13.53650
H	11.59630	3.00750	13.14960
H	10.46390	2.76970	14.46860
H	13.64250	2.62470	14.76870
H	12.45890	2.29150	16.01950
H	13.68900	3.50910	16.28970
H	14.42120	6.26310	12.35550
H	15.37520	4.14300	11.64100
H	15.49790	4.24660	13.38970
H	14.25900	3.23410	12.64440
H	12.19420	6.14140	11.29710
H	12.20480	4.38680	11.43720
H	13.38020	5.19770	10.41840

H	11.84140	9.63640	15.60220
H	13.01830	10.96390	17.28730
H	13.17570	13.04400	18.62160
H	11.49420	14.82240	18.35840
H	9.62570	14.55230	16.75610
H	8.48270	13.18820	15.07670
H	2.75660	13.32940	12.95070
H	1.39630	14.77190	14.40300
H	2.81640	15.67270	14.91000
H	2.25970	15.76370	13.24450
H	3.90420	12.00700	14.67540
H	3.90670	13.38030	15.77480
H	2.39030	12.63290	15.29480
H	4.95830	16.70000	13.77800
H	5.01870	15.42360	15.89320
H	6.65990	14.88440	15.55200
H	6.32780	16.58580	15.85220
H	7.35780	17.32590	13.69470
H	7.78820	15.67980	13.24670
H	6.82860	16.67550	12.15540
H	5.79420	14.95730	10.75450
H	4.29700	13.73180	9.23150
H	4.94040	12.65190	10.45420
H	3.28120	13.23360	10.57740
H	3.97970	16.22650	9.73790
H	4.25920	16.84680	11.35480
H	2.87530	15.80060	11.03270
H	6.06310	10.37750	11.56830
H	4.89870	9.05100	9.88160
H	2.90110	7.47610	9.68240
H	1.03440	7.15190	8.11720
H	-0.81960	5.37800	2.29060
H	-3.77710	3.91400	-0.44950
H	-4.28900	1.65900	-0.39530
H	-2.80120	1.55830	-1.34130
H	-2.75930	1.27840	0.40410
H	-2.88360	5.65990	-1.45220
H	-3.64830	9.67340	-0.36290
H	-3.72930	11.44720	-2.04940
H	-3.78400	10.23050	-3.32630
H	-5.15300	10.41870	-2.22300
H	-1.60220	9.22380	0.56720
O	-2.34810	5.55400	4.62030
C	-3.37360	5.12960	4.08860
N	-3.81820	3.88210	4.27170
C	-3.04520	2.77250	4.81740
C	-2.97650	1.61740	3.80480
C	-2.00980	0.51740	4.21010
C	-0.77920	0.39780	3.56330
C	0.11090	-0.60290	3.94570
C	-0.21700	-1.49270	4.97530
C	0.73790	-2.55490	5.39530
C	1.38960	-3.34710	4.44640
C	2.27930	-4.35030	4.82610
C	2.54360	-4.58660	6.19630
C	3.43100	-5.58930	6.63220
C	3.72320	-5.85140	8.00870
C	4.62850	-6.87760	8.43840
C	5.28960	-7.72140	7.43020

C	5.85850	-8.44300	6.62560
Si	6.75590	-9.55710	5.47820
C	7.87100	-10.69770	6.53320
C	8.96670	-9.93450	7.30210
C	7.07130	-11.58770	7.50510
C	5.46060	-10.61760	4.54770
C	4.58190	-9.81660	3.57010
C	6.04660	-11.86050	3.84940
C	7.84130	-8.48830	4.31560
C	8.56050	-9.29910	3.22300
C	7.11490	-7.27580	3.69930
C	4.90800	-7.10640	9.82720
C	5.81140	-8.11600	10.28920
C	6.09830	-8.35120	11.64700
C	7.00090	-9.36060	12.06590
C	7.25470	-9.55510	13.42230
C	6.62660	-8.76300	14.37880
C	5.73450	-7.76370	13.99460
C	5.45830	-7.54400	12.62170
C	4.56600	-6.54680	12.18450
C	4.26230	-6.29460	10.80880
C	3.34970	-5.27390	10.37990
C	2.68000	-4.44210	11.39370
C	2.14080	-3.76420	12.25500
Si	1.36620	-2.76280	13.58480
C	-0.49680	-3.19420	13.72810
C	-1.11070	-2.81680	15.09050
C	-0.83120	-4.66050	13.38730
C	2.28550	-3.12750	15.22280
C	2.21900	-4.60750	15.64710
C	3.75140	-2.65280	15.20360
C	1.58150	-0.92160	13.11590
C	0.93610	-0.55500	11.76520
C	1.12120	0.05750	14.21170
C	3.07460	-5.04220	8.99100
C	2.17420	-4.03090	8.52970
C	1.89590	-3.78860	7.17100
C	0.99600	-2.77800	6.75180
C	-1.45860	-1.36790	5.60890
C	-2.35260	-0.37060	5.22930
C	-4.15440	5.97650	3.11670
C	-4.81960	5.39130	2.02940
C	-5.45340	6.17030	1.05610
C	-6.12140	5.49740	-0.11270
N	-7.22480	6.08720	-0.58710
C	-8.00900	5.59790	-1.71450
O	-5.67360	4.44420	-0.56720
C	-5.40730	7.56550	1.17400
C	-4.75420	8.17690	2.25190
C	-4.66960	9.67710	2.31280
N	-4.73460	10.22500	3.53170
C	-4.62560	11.65180	3.80470
O	-4.55950	10.33890	1.27990
C	-4.13660	7.37270	3.22200
H	-4.80340	3.71010	4.08300
H	-3.42850	2.43310	5.78130
H	-2.02080	3.07700	5.05320
H	-3.96960	1.19230	3.65260
H	-2.68130	2.00720	2.82890

H	-0.51060	1.07890	2.76630
H	1.06590	-0.68040	3.44610
H	1.19440	-3.19020	3.39530
H	2.76990	-4.95470	4.07670
H	3.91030	-6.18240	5.86790
H	8.38050	-11.36340	5.83550
H	8.53770	-9.22620	8.01250
H	9.61590	-9.37690	6.62690
H	9.60560	-10.61650	7.86470
H	7.72390	-12.27930	8.03940
H	6.54410	-10.99180	8.25170
H	6.32890	-12.18910	6.98000
H	4.79070	-10.99230	5.32300
H	4.16620	-8.92580	4.04290
H	5.14540	-9.50140	2.69180
H	3.74190	-10.41550	3.21570
H	6.71650	-11.59280	3.03310
H	5.25630	-12.48210	3.42610
H	6.60470	-12.48800	4.54410
H	8.62020	-8.06840	4.95270
H	9.29400	-8.68670	2.69680
H	9.09490	-10.15160	3.64350
H	7.86030	-9.67380	2.47640
H	6.65710	-6.64950	4.46610
H	6.33150	-7.57650	3.00530
H	7.80950	-6.64180	3.14640
H	6.31520	-8.74460	9.57070
H	7.49080	-9.97690	11.32570
H	7.94430	-10.32620	13.73470
H	6.83350	-8.92520	15.42700
H	5.24930	-7.15020	14.74010
H	4.09540	-5.94720	12.94840
H	-1.00160	-2.58840	12.97500
H	-2.19080	-2.96990	15.09310
H	-0.69660	-3.41990	15.89900
H	-0.93670	-1.77070	15.33810
H	-0.50760	-4.92100	12.37880
H	-0.35360	-5.35700	14.07620
H	-1.90570	-4.84330	13.43120
H	1.77760	-2.55240	15.99800
H	1.19020	-4.94300	15.77290
H	2.69240	-5.25730	14.91010
H	2.72360	-4.77060	16.60030
H	4.23760	-2.82360	16.16510
H	4.33180	-3.17940	14.44430
H	3.82450	-1.58490	14.99580
H	2.65570	-0.77270	12.99570
H	1.16540	0.47380	11.48380
H	1.30380	-1.19480	10.96190
H	-0.14970	-0.65020	11.79700
H	1.40420	1.08200	13.96580
H	1.57360	-0.17620	15.17590
H	0.03840	0.04440	14.33460
H	1.66930	-3.40530	9.25000
H	0.51240	-2.16780	7.50010
H	-1.73510	-2.05490	6.39560
H	-3.30720	-0.29070	5.72880
H	-4.81880	4.31460	1.91490
H	-7.59750	6.88360	-0.07890

H	-8.16490	6.40340	-2.43180
H	-7.52830	4.77110	-2.24040
H	-8.98450	5.25670	-1.36780
H	-5.86110	8.18690	0.41160
H	-4.97800	9.62970	4.31600
H	-3.99550	11.81200	4.67940
H	-4.19040	12.20680	2.97160
H	-5.61160	12.06580	4.01480
H	-3.59290	7.83200	4.03830

6. Author contributions

G. L. and E. W. M. conceived the project and designed the research. G. L. designed the target compounds, the self-assembly experiments and the steady-state/time-resolved emission measurements and analyzed the resulting data. G.L. and M.B. (with supervision from G. L.) synthetized the target BTA-Pnc molecules, performed the absorption, CD, emission and FT-IR measurements and prepared the samples for AFM studies. G. L., M. B. and S. C. J. M. conducted the TCSPC measurements. G. L. and A. S. prepared the solutions for transient absorption spectroscopy measurements. A. S. conducted the pump-probe experiments and performed the global fitting and Monte Carlo simulation of transient data. S. A. H. J. performed the computational modeling of supramolecular polymerization. E. W. M., R. H. F. and A. R. supervised the research and acquired funding. G. L. led the writing of the manuscript with substantial contributions from A. S. All authors discussed the results, revised the manuscript and gave approval to the final version of the manuscript.

7. References

(1) Brunsveld, L.; Schenning, A. P. H. J.; Broeren, M. A. C.; Janssen, H. M.; Vekemans, J. A. J. M.; Meijer E. W. Chiral Amplification in Columns of Self-Assembled *N,N',N''*-Tris((*S*)-3,7-dimethyloctyl)benzene-1,3,5-tricarboxamide in Dilute Solution. *Chem. Lett.* **2000**, *29* (3), 292-293.

(2) Masuda, M.; Jonkheijm, P.; Sijbesma, R. P.; Meijer, E. W. Photoinitiated Polymerization of Columnar Stacks of Self-Assembled Trialkyl-1,3,5-benzenetricarboxamide Derivatives. *J. Am. Chem. Soc.* **2003**, *125* (51), 15935-15940.

(3) Roosma, J.; Mes, T.; Leclère, P.; Palmans, A. R. A.; Meijer, E. W. Supramolecular Materials from Benzene-1,3,5-Tricarboxamide-Based Nanorods. *J. Am. Chem. Soc.* **2008**, *130* (4), 1120-1121.

(4) Zhao, D. H.; Moore, J. S. Nucleation–elongation: a mechanism for cooperative supramolecular polymerization. *Org. Biomol. Chem.* **2003**, *1* (20), 3471-3491.

(5) Walker, B. J.; Musser, A. J.; Beljonne, D.; Friend, R. H. Singlet Exciton Fission in Solution. *Nat. Chem.* **2013**, *5* (12), 1019-1024.

(6) Sharma, A. ; Athanasopoulos, S.; Kumarasamy, E.; Phansa, C.; Asadpoordarvish, A.; Sabatini, R. P.; Pandya, R.; Parenti, K. R.; Sanders, S. N.; McCamey, D. R.; Campos, L. M.; Rao, A.; Tayebjee, M. J. Y.; Lakhwani, G. Pentacene–Bridge Interactions in an Axially Chiral Binaphthyl Pentacene Dimer. *J. Phys. Chem. A* 2021, *125* (33), 7226-7234.
